# Asperopiperazines A and B: Antimicrobial and Cytotoxic Dipeptides from a Tunicate-Derived Fungus *Aspergillus* sp. DY001

**DOI:** 10.3390/md20070451

**Published:** 2022-07-10

**Authors:** Diaa T. A. Youssef, Lamiaa A. Shaala, Grégory Genta-Jouve

**Affiliations:** 1 Department of Natural Products, Faculty of Pharmacy, King Abdulaziz University, Jeddah 21589, Saudi Arabia; 2 Natural Products Unit, King Fahd Medical Research Center, King Abdulaziz University, Jeddah 21589, Saudi Arabia; lamiaa.elnady@med.suez.edu.eg; 3 Department of Medical Laboratory Sciences, Faculty of Applied Medical Sciences, King Abdulaziz University, Jeddah 21589, Saudi Arabia; 4 UAR3456 CNRS LEEISA, Laboratoire Ecologie, Evolution, Interactions des Systèmes Amazoniens, Centre de Recherche de Montabo, IRD, 275 Route de Montabo, CEDEX BP 70620, 97334 Cayenne, France; gregory.genta-jouve@cnrs.fr

**Keywords:** red sea tunicate, *Didemnum* sp., associated fungi, *Aspergillus* sp. DY001, marine dipeptides, asperopiperazines A and B, antimicrobial activity, cell lines’ growth inhibition

## Abstract

Investigation of the cytotoxic fractions of the ethyl acetate extract of the fermentation broth of the tunicate-derived *Aspergillus* sp. DY001 afforded two new dipeptides, asperopiperazines A and B (**1** and **2**), along with the previously reported compounds (+)-citreoisocoumarin (**3**) and (−)-6,8-di-*O*-methylcitreoisocoumarin (**4**). Analyses of the 1D and 2D NMR spectroscopic data of the compounds supported their structural assignments. Asperopiperazine A (**1**) is a cyclic dipeptide of leucine and phenylalanine moieties, which are substituted with an *N*-methyl and an *N*-acetyl group, respectively. On the other hand, asperopiperazine B (**2**) is a cyclic dipeptide of proline and phenylalanine moieties with a hydroxyl group at C-2 of the proline part. The absolute configuration of the amino acid moieties in **1** and **2** were determined by Marfey’s analyses and DFT NMR chemical shift calculations, leading to their assignment as cyclo(l-*N*Me-Leu-l-*N*Ac-Phe) and cyclo(d-6-OH-Pro-l-Phe), respectively. Asperopiperazines A and B displayed higher antimicrobial effects against *Escherichia coli* and *Staphylococcus aureus* than *Candida albicans.* Furthermore, compounds **1**–**4** displayed variable growth inhibitory effects towards HCT 116 and MDA-MB-231 cells, with asperopiperazine A as the most active one towards HCT 116.

## 1. Introduction

Secondary metabolites played an important and inspiring role in the process of drug discovery and development [1,2,3,4,5]. Tunicates (ascidians) are well-known marine invertebrates and represent an extraordinary and major source of secondary metabolites with pharmaceutical and biomedical importance, including the marketed drugs Trabectedin and Plitidepsin [5].

Among the tunicates, the genus *Didemnum* belongs to the most well-known family (Didemnidae), and includes the most investigated species among the tunicates. In a recent review, more than 212 compounds were reported from 69 species of the genus *Didemnum* worldwide [5]. The compounds displayed variable anticancer, antimicrobial, antiviral and antiprotozoal activities, and others affecting the central nervous system. The most prominent members of these secondary metabolites of the *Didemnum* are the lamellarins, which have been widely studied for their anticancer potential [6]. In addition, the *Didemnum*-derived enediynes, namenamicin and shishijimicins, are exceptionally anticancer-potent at the picomolar level [7]. Moreover, the structurally correlated enediynes have been approved by the FDA for their antibody-drug conjugates [8]. Vitilevuamide, an unusual peptide, showed a potent effect against different cancer cell lines at the sub-nanomolar level [9]. Finally, divamide A, a cyclic peptide, inhibited the replication of HIV at ≈200 nM in cell lines [10].

Filamentous fungi are capable of biosynthesizing secondary metabolites possessing diverse chemical entities and biomedical potential, which are often non-existent in other domains of life. The examples of pharmaceuticals from fungi include the mechanistically unique immunosuppressant, cyclosporin [11], the top-selling antihypercholesteremic statins [12], and the antibiotic penicillins and cephalosporins [13]. In addition to these well-known compounds, numerous fungal products are in clinical development, or find use as biological probes. Many fungal products, such as aflatoxins [14], ergot alkaloids [15], and trichothecenes [16], are agriculturally important toxins, and some of these also have use as lead drug structures. Filamentous fungi have thus been key organisms in drug discovery and biotechnology.

The marine-derived fungi represent an essential and rich source of secondary metabolites that are useful for the development of future drugs. The secondary metabolites associated with marine fungi include diverse chemical classes, such as coumarins, polyketides, alkaloids, acetophenones, polyphenols, and xanthones, and many others [17,18,19,20,21].

In a recent review summarizing the chemical and biological diversity of the secondary metabolites from the marine-derived genus *Aspergillus*, the major reported classes included anthracene and biphenyl derivatives, isoprenoids, alkaloids, butyrolactones, and others [22]. From the almost 2000 secondary metabolites reported from the members of the marine-derived *Aspergillus* genus, 332 compounds were associated with different bioactivities [22]. Among the ophiopolines, biphenyl derivatives, and alkaloids, 30% of these compounds showed exceptional anticancer activities and 27% of these compounds displayed anti-inflammatory and antimicrobial activities. On the other hand, 10% of the compounds possessed an anti-diabetic effect, 8% showed anti-inflammatory effects, 8% possessed antioxidant properties, 4% of the compounds displayed antifungal effects, while 4% of the compounds showed antiviral activity. Finally, 9% of the compounds including biphenyl derivatives, butenolides, diterpenes, xanthones, diketopiperazines, and steroids, possessed other biological activities [22].

Plinabulin, a synthetic analogue of the *Aspergillus*-derived diketopiperazine halimide, was found to target the tubulin network. The mechanism of action of plinabulin on tubulin is different from the effect of the *Vinca* alkaloids and taxanes. Plinabulin inhibits the tubulin network polymerization by interacting with the colchicine-binding domain of β-tubulin [23,24]. Plinabulin was recently FDA-approved for the treatment of non-small lung cancer (advanced metastatic) [25,26].

As a continuation of our work to uncover the biologically active compounds from marine microbes [27], the cytotoxic fractions of the ethyl acetate extract of the culture of the tunicate-derived fungus *Aspergillus *species DY001 were investigated. As a result, two cyclic dipeptides, asperopiperazines A (**1**) and B (**2**), together with (+)-citreoisocoumarin (**3**) [28,29] and (−)-6,8-di-*O*-methylcitreoisocoumarin (**4**) [30] were purified from the active fractions of the extract and their structures were characterized. In this report, we describe the structural assignments, the antimicrobial activities against several pathogens, and the growth inhibitory effects of the compounds against three cancerous cell lines.

## 2. Results and Discussion

### 2.1. Structure of Asperopiperazine A *(**1**)*

**Figure 1:**
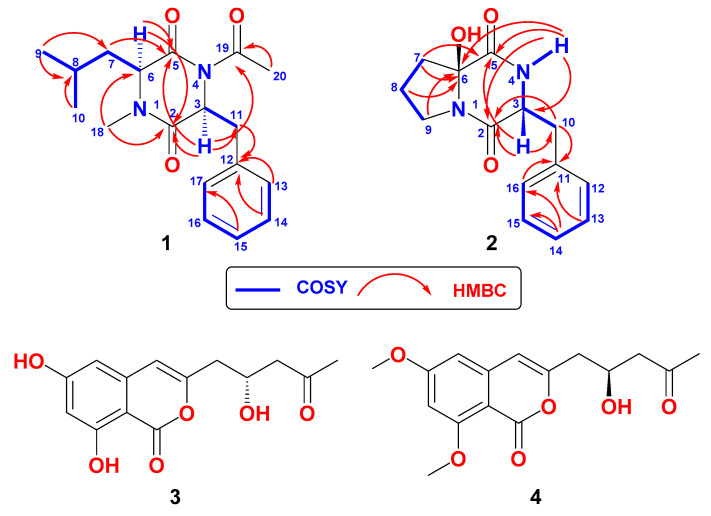
Structures of **1**–**4**, and COSY and HMBC correlations of **1** and **2**.

Compound **1** (Figure 1) was isolated as a yellow oil with molecular formula C_18_H_24_N_2_O_3_, as established from the HRESIMS pseudomolecular ion peak at *m*/*z* 339.1683 (calc. for C_18_H_24_N_2_NaO_3_ [M + Na]^+^, 339.1679) (Figure S1, Supplementary Materials). Analyses of the 1D and 2D NMR spectra of 1 (Figures S2–S7, Supplementary Materials) supported the assignment of its structure. The ^13^C NMR of **1** (Table 1) displayed signals corresponding to the 18 carbons, classified to four methyls, two methylenes, three aliphatic methines, five aromatic methines, and four quaternary carbons, as supported by the ^13^C NMR and HSQC experiments (Table 1). The existence of a 2,5-diketopiperazine [31] backbone in **1** was obvious from the ^1^H and ^13^C NMR signals at δ_H/C_ 174.5 (C, C-2), 175.2 (C, C-5), and the methine signals at δ_H_/δ_C_ 5.00 (t, *J* = 7.28 Hz, H-3)/52.6 (CH, C-3) and 5.04 (dd, *J* = 9.6, 6.0 Hz, H-6)/56.3 (CH, C-6). In addition to these signals, the remaining ^1^H and ^13^C signals of **1** supported the presence of the *N*-acetylphenylalanine and *N*-methylleucine units in **1**. The presence of the ^1^H-^1^H and ^1^*J* ^1^H-^13^C couplings in the COSY and HSQC of **1** from H-3 to H_2_-11 at δ_H/C_ 3.06 (dd, *J* = 13.2, 7.6 Hz), 2.93 (dd, *J* = 13.2, 7.2 Hz)/38.7 (CH_2_, H_2_-11/C-11), and from H-13 to H-17 at δ_H/C_ 7.25 (2H, m)/129.6 (2 x CH, H-13,17/C-13,17), 7.25 (m, 2H)/130.4 (2 x CH, H-14,16/C-14,16), 7.21 (m)/128.1 (CH, H-15/C-15) along with the quaternary signal at 138.0 (C, C-12) supported the assignment of a phenylalanine moiety. The presence of the signals at δ_H/C_ 172.8 (C, C-19), 1.89 (3H, s)/22.1 (CH_3_, C-20) supported the presence of an acetyl moiety in **1**. The presence of the acetyl moiety at *N*-4 was supported from the HMBC correlations from H-3 (δ_H_ 3.06) to C-19 (δ_C_ 172.8) and from H_3_-20 (δ_H_ 1.89) to C-19 (Table 1 and Figure 1). Accordingly, the existence of the *N*-acetylphenylalanine moiety in **1** was assigned and confirmed.

Similarly, the presence of *N*-methylleucine unit in **1** was assigned from the ^1^H-^1^H and ^1^*J* ^1^H-^13^C correlations in the COSY and HSQC experiments of **1** and its ^1^H and ^13^C NMR resonances at δ_H/C_ 175.2 (C, C-5, 5.04 (dd, *J* = 9.6, 6.0 Hz, H-6)/56.3 (CH, C-6), 1.62 (m, H_2_-7)/38.1 (CH_2_, C-7), 1.42 (m, H-8)/24.5 (CH, C-8), 0.91 (d, *J* = 6.6 Hz, H_3_-9)/23.6 (CH_3_, C-9) and 0.85 (d, *J* = 6.6 Hz, H_3_-10)/21.0 (CH_3_, C-10) supported the presence of the leucine moiety in **1** (Figure 1). In addition, the ^1^H/^13^C NMR signal at δ_H_/δ_C_ 2.84 (3H, s, H_3_-18/31.7 (CH_3_, C-18) was assigned as *N*1-Me. Thus, the *N*-methylleucine moiety was assigned. HMBC from H_3_-18 (NCH_3_) to C-2 (δ_C_ 174.5, C) and C-6 (δ_C_ 56.3, CH) supported the placement of the methyl group at N-1 (Table 1 and Figure 1).

**Figure 2:**
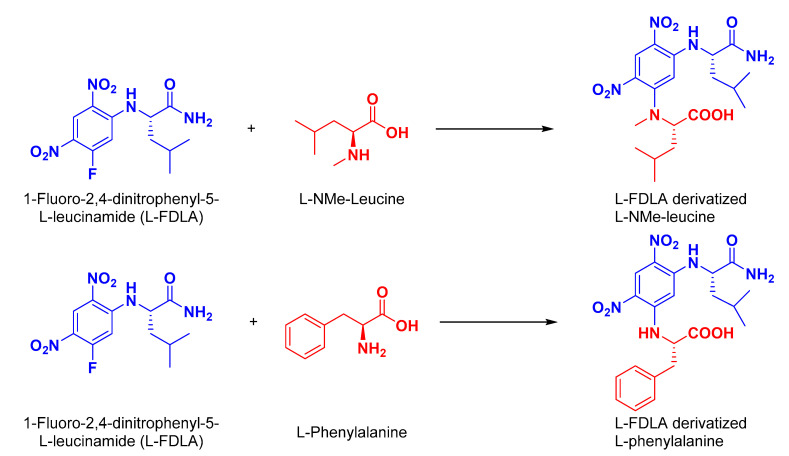
Reaction of l-FDLA with hydrolytic products of asperopiperazine A (**1**).

The absolute configuration of the amino acid components of **1** was determined by Marfey’s analysis [32]. After the treatment of **1** in 6 M HCl at 110 °C for 16 h, the hydrolysate was derivatized with 1-fluoro-2,4-dinitrophenyl-5-l-leucinamide (l-FDLA), and the retention times were compared with the FDLA derivatives of the standard amino acids phenylalanine and *N*-methylleucine (both d and l). The amino acids in **1** were found to possess the l-configuration. Figure 2 shows the reaction of l-FDLA with the hydrolytic products of **1**. Accordingly, **1** was assigned as cyclo(l-*N*Me-Leu-l-*N*Ac-Phe) and named asperopiperazine A.

### 2.2. Structure of Asperopiperazine B *(**2**)*

Asperopiperazine B (**2**) (Figure 1) with the molecular formula of C_14_H_16_N_2_O_3_, as supported by the HREISMS pseudomolecular ion peak at *m*/*z* 283.1055 (calcd for C_14_H_16_N_2_O_3_Na, [M + Na]^+^, 283.1053) (Figure S8, Supplementary Materials). Interpretation of the 1D and 2D NMR (Figures S16–S20, Supplementary Materials) spectra of **2** supported its planar structure. The ^13^C NMR spectrum along with the HSQC showed signals for 14 carbons, which were classified into five aromatic methines, one aliphatic methine, four methylenes, and four quaternary carbons (Table 1). Again, the characteristic resonances for a cyclic dipeptide backbone [31] were noticed in **2**. These included two carbonyls at δ_C_ 166.1 (C, C-2) and 167.9 (C, C-5), a methine attached to heteroatom at δ_H_/δ_C_ 4.45 (dd, *J* = 11.0, 3.3 Hz, H-3)/55.8 (CH, C-3), and an interchangeable signal at δ_H_ 5.59 (1H, brs, N*H*-4). Moreover, the remaining ^1^H and ^13^C NMR in **2** supported the presence of a proline nucleus, which is substituted at C-2 (C-6 in **2**) [31], and a phenylalanine part (Table 1).

Together with the quaternary amidic carbon at δ_C_ 167.4 (C, C-5), the ^1^H and ^13^C NMR resonances at δ_H/C_ 87.6 (C, C-6), 2.23 (1H, m, H-7a), 2.17 (1H, m, H-7b)/36.8 (CH_2_, C-7), 2.17 (1H, m, H-8a), 2.00 (1H, m, H-8b)/19.9 (CH_2_, C-8), and 3.79 (1H, m, H-9a), 3.59 (1H, m, H-9b)/45.5 (CH_2_, C-9) and 2.98 (1H, brs, O*H*) constitute a two-substituted proline moiety [31]. The ^1^H-^1^H COSY experiment supported the assignment of the substituted proline part from consecutive ^3^*J* vicinal couplings from H_2_-7 to H_2_-8 and from H_2_-8 and H_2_-9. The absence of any further coupling within the proline part suggested the quaternary nature of C-6 [31]. The downfield shift of C-6 at δ_C_ 87.6 [31] together with the one-proton interchangeable signal at δ_H_ 2.99 (OH) supported the existence of a hydroxyl moiety at C-6 [31]. Finally, HMBC correlations of H_2_-8/C-6 (δ_C_ 87.6), H_2_-7/C-6, and H_2_-9/C-6 supported the assignment of 6-OH-proline moiety (Table 1 and Figure 1).

Similarly, the existence of a phenylalanine part in **2** was supported from the amidic carbonyl ^13^C NMR resonances at δ_C_ 166.1 (C, C-2), together with the COSY and HSQC experiments and ^1^H and ^13^C NMR signals at δ_H/C_ 5.59 (brs, N*H*), 4.45 (1H, dd, *J* = 11.0, 3.3 Hz, H-3)/55.8 (CH, C-3), 3.63 (1H, dd, *J* = 14.4, 4.2 Hz, H-10a), 2.74 (1H, dd, *J* = 14.4, 10.2 Hz, H-10b)/36.9 (CH_2_, C-10), 135.7 (C, C-11), 7.23 (2H, d, *J* = 7.5 Hz, H-12,16)/129.1 (2 x CH, C-12,16), 7.35 (2H, t, *J* = 7.5 Hz, H-13,15)/129.3 (2 x CH, C-13,15), and 7.29 (1H, t, *J* = 7.5 Hz, H-14)/127.7 (CH, C-14).

The vicinal coupling between the N*H* signal (δ_H_ 5.58) and H-3 (δ_H_ 4.45), which further couples with H-10a (δ_H_ 3.63) and H-10b (δ_H_ 2.74), as well as the consecutive couplings within the aromatic moiety from H-12 to H-16 secured the assignment of the phenylalanine moiety in **2**. Further, the connection of the proline part with the phenylalanine parts was also supported by HMBC from N*H* (δ_H/_5.58) to C-6 (δ_C_ 87.6) (Table 1 and Figure 1).

**Figure 3:**
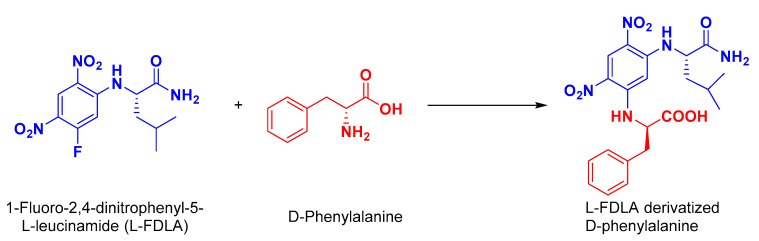
Reaction of l-FDLA with hydrolytic products of asperopiperazine B (**2**).

Similar to **1**, the absolute configuration of the phenylalanine moiety in **2** was determined by Marfey’s analysis [31]. After treatment of **2** in 6 M HCl at 110 °C for 16 h, the hydrolysate was derivatized with 1-fluoro-2,4-dinitrophenyl-5-l-leucinamide (l-FDLA), and the retention times were compared with the l-FDLA derivatives of the standard amino acids d- and l-Phe. The derivatized amino acid in **2** was found to possess the d-configuration. Thus, the existence of d-Phe unit was confirmed in **2**. Figure 3 displays the reaction of l-FDLA with the hydrolytic product of **2**.

Attempts to confirm the configuration of C-6 of the proline part by Marfey’s method were impossible, due to the decomposition of **2** under acidic conditions.

To assign the absolute configuration of the OH moiety at C-6 of compound **2**, the ^13^C NMR chemicals shifts of the 6-OH proline moiety in **2** with cyclo(d-6-OH-Pro-l-Phe) and cyclo(l-6-OH-Pro-l-Phe) were compared. As shown in Table 2, the ^13^C shifts of **2** are perfectly matching those of cyclo(d-6-OH-Pro-l-Phe) [33]. Thus, the d-configuration was assigned for the proline moiety in **2**.

**Figure 4:**
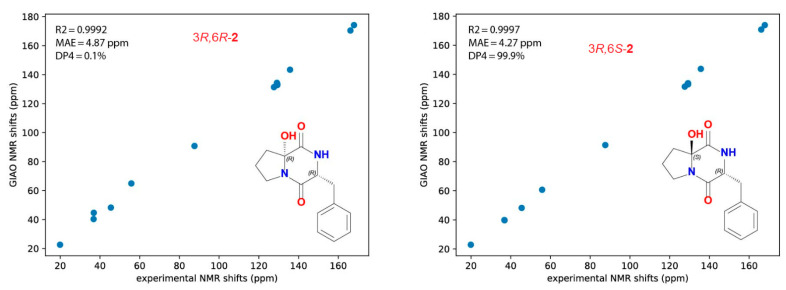
Calculated and predicted ^13^C NMR chemical shifts for **2**.

As shown in Figure 4, the 3*R*,6*S* configurations for **2** were supported by all of the metrics used. The correlation between the experimental and theoretical chemical shifts was higher for 3*R*,6*S* (R2 = 0.9997) and the mean average error (MAE) value was lower for 3*R*,6*S* isomer and, finally, a 99.9% DP4 score for this isomer (Figure 4). All of the calculations for both isomers (3*R*,6*R* and 3*R*,6*S*) of **2** can be found in Table S1 of the Supplementary Materials. Accordingly, **2** was assigned as cyclo(d-6-OH-Pro-d-Phe) and named asperopiperazine B.

Compound **3** possesses molecular formula C_14_H_14_O_6_ as supported from (+)-HRESIMS (Figure S15, Supplementary Materials). Its structure was assigned by interpretation of its 1D and 2D NMR spectra (Figures S16–S20, Supplementary Materials). The ^1^H and ^13^C NMR data of **3** are similar to those reported for (+)-citreoisocoumarin [28,29]. In addition, the optical rotation value of compound **3** was [α]_D_ = +43.5°, which was in good agreement with the reported value ([α]_D_ = +46.5°) for (+)-citreoisocoumarin [28]. Thus, **3** was assigned as (+)-citreoisocoumarin.

Compound **4** possesses the molecular formula C_16_H_18_O_6_, as supported from (+)-HRESIMS (Figure S21, Supplementary Materials). Its structure was assigned by interpretation of its 1D and 2D NMR spectra (Figures S22–S26, Supplementary Materials). Its ^1^H and ^13^C NMR data are similar to those reported for (−)-6,8-di-*O*-methylcitreoisocoumarin [30]. Furthermore, the optical rotation value of compound **4** was [α]_D_ = −10.5°, which was in good agreement with the reported value ([α]_D_ = −10°) for (−)-6,8-di-*O*-methylcitreoisocoumarin [30]. Thus, **4** was assigned as (−)-6,8-di-*O*-methylcitreoisocoumarin.

### 2.3. Biological Activities of the Compounds

The antimicrobial effects of **1**–**4** were determined in a disc diffusion assay at a concentration of 50 µg/disc against three organisms. Compounds **1** and **2** showed moderate antibacterial effects towards *E. coli* (inhibition zones = 17–22 mm) and *S. aureus* (inhibition zones = 16–18 mm) and a lower effect against *C. albicans (*inhibition zones = 11–12 mm), suggesting their higher effects towards *E. coli* and *S. aureus* (Table 3).

On the other hand, compound **3** displayed a higher activity against *S. aureus* (inhibition zone of 19 mm) than *C. albicans* (17 mm) and *E. coli* (11 mm). On the contrary, compound **4** was less active than **3** (inhibition zones 6–9 mm) against these microbes. This difference in the activity between **3** and **4** could be attributed to the lack of the phenolic moieties in **4**.

Furthermore, **1** and **2** displayed minimum inhibitory concentration (MIC) values of 8 and 4 μM against *E. coli*, and 8 and 8 μM against *S. aureus*, respectively. On the contrary, compound **3** was active against *C. albicans* with MIC value of 8 μM (Table 3).

Using MTT assay, compounds **1**–**4** displayed their main activity against HCT 116 cells with IC_50_ values of 15.1–19.3 μM with asperopiperazine A (**1**) as the most active compound (Table 4). On the contrary, the compounds showed weak activities towards the MDA-MB-231 (IC_50_ = 24.3–35.0 μM), while they were inactive against HeLa cells (≥50.0 μM). These data suggest that **1**–**4** possess better selectivity towards HCT 116 than MDA-MB-231 and weak effect towards HeLa cells.

## 3. Materials and Methods

### 3.1. General Experimental Procedures

Optical rotations were acquired on a digital DIP-370 polarimeter (JASCO, Oklahoma City, OK, USA). The IR spectra were recorded on a Shimadzu Infrared-400 spectrophotometer (Shimadzu, Kyoto, Japan). One- and two-dimensional NMR spectra were acquired on Bruker Avance DRX 600 MHz (600 MHz for ^1^H and 150 MHz for ^13^C NMR) (Bruker, Rheinstetten, Germany) or on Bruker Ascend 850 MHz (850 MHz for ^1^H and 213 MHz for ^13^C NMR) (Bruker BioSpin, Billerica, MA, USA) spectrometers using DMSO-*d6*, CDCl_3_, and CD_3_OD as solvents. NMR spectra were referenced to the residual protonated solvent signals (DMSO-*d_6_*: 2.49 ppm for ^1^H and 39.5 ppm for ^13^C; CHCl_3_: 7.26 ppm for ^1^H and 77.0 ppm for ^13^C, CH_3_OH: 3.30 ppm for ^1^H and 49.0 ppm for ^13^C). The positive ion HRESIMS data were obtained with a Micromass Q-ToF equipped with leucine enkephalin lock spray, using *m*/*z* 556.2771 [M + H]^+^ as a reference mass. Sephadex LH-20 (0.25–0.1 mm, Pharmacia) was used for the column chromatography. Precoated silica gel 60 F-254 plates (Merck) were used for TLC.

### 3.2. The Host Organism, Didemnum Species

**Figure 5:**
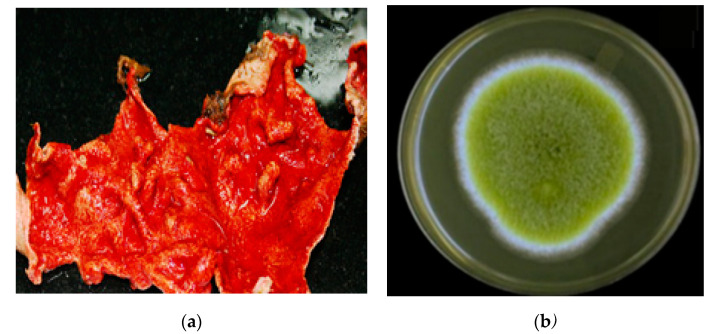
(**a**) Photographs of the Red Sea tunicate *Didemnum* sp. and (**b**) the fungus *Aspergillus* sp. DY001.

The tunicate *Didemnum* species (Figure 5) was collected by hand at a depth of −10–13 m using scuba diving off Jizan, at the Saudi Red Sea coast. The identification of the specimen was provided by Dr. Francois Monniot at the National Museum of Natural History (MNHN), Paris. A specimen of the tunicate was deposited in our invertebrates’ collection at the Department of Natural Products at King Abdulaziz University under code no. DY31.

### 3.3. Purification of the Fungal Isolate

The fungal isolate DY001 (Figure 5) was purified from the internal tissue of the *Didemnum* species, as described earlier [34]. In brief, surface sterilization of the tissue of the *Didemnum* species was performed using 70% EtOH. A small piece of the internal tissue of the tunicate was mixed with sterile seawater (10 mL). The homogenized tissue of the tunicate was diluted serially to 1:10, 1:100, and 1:1000. About 100 μL of each dilution was used for culturing the isolate on Sabouraud dextrose agar medium [34]. The medium was prepared in sterile seawater containing 0.25% chloramphenicol (w/v). The cultured plates were stored for 1–2 weeks at 30 °C until complete growth of the fungal isolate. Subsequent purification steps were carried out until a pure strain was obtained.

### 3.4. Purification of gDNA from Fungal Isolates

The fungal isolate was cultured in Sabouraud dextrose broth at 28 °C for 3–4 days. The mycelia were separated from the broth and freeze-dried separately. The fungal gDNA was extracted using the DNA Mini Kit QIAamp, according to the instructions of the manufacturer and as reported earlier [34].

### 3.5. Fungal ITS-rDNA Fragments’ Amplification

The gDNA of the fungal strain DY001 represented the template for the amplification of the Internal Transcribed Spacer-rDNA (ITS-rDNA) fragments using the primers ITS1 and ITS4 [34,35]. The mixture for amplification and the conditions of the PCR were used as previously described [34,35].

### 3.6. Sequence of ITS-rDNA Fungal Region

The sequence of the ITS-rDNA fungal region of the isolate was used for the initial characterization through comparing it with related sequences in the NCBI database [36]. The Clustal X (Version 1.83) program [37] was used to edit and align the fungal ITS-rDNA sequence with the best n-BLAST hits from GenBank [38]. Further manual adjustments were carried out, using BioEdit software [38]. The base composition of the fungal sequences was calculated, using the MEGA v.6 program [39].

### 3.7. Characterization of the Fungal Isolate

The sequence analyses of the fungal isolate DY001 displayed 99.5% sequence similarity with *Aspergillus flavipes* (KF986416) in BLAST. This sequence was placed in the GenBank under Accession Numbers MN818770 and it was released on 17 December 2019.

### 3.8. Large-Scale Culture of Aspergillus sp. DY001

A culture of five-liter fermentation broth of the fungus was prepared in Sabouraud Dextrose broth amended by 3% NaCl (w/v) by shaking for 15 days at 30 °C and 180 rpm in a shaker incubator. Later, each flask of the culture broth was partitioned with 200 mL of EtOAc three times at room temperature. The combined EtOAc extracts were evaporated under reduced pressure and the resulting extract was used for further purification of the compounds.

### 3.9. Purification of Compounds ***1***–***4***

The crude ethyl acetate extract (1.67 g) was partitioned on a VLC silica column using hexane-EtOAc-MeOH gradients, using 50% hexane in EtOAc, 100% EtOAc, 5% MeOH in EtOAc, 30% MeOH in EtOAc, 50% MeOH in EtOAc, 70% MeOH in EtOAc, 80% MeOH in EtOAc, and 100% MeOH. The polar fraction eluted with 5% MeOH (360 mg) was partitioned on Sephadex LH-20 with MeOH-CH_2_Cl_2_ (1:1) to afford five fractions (A–E). The cytotoxic fraction (Fr. E, 130 mg) was subjected to partition on Sep-Pak C18 Cartridge (Waters, 10 g) using H_2_O-MeOH gradients to give six main fractions. The fraction eluted with 70% MeOH in EtOAc from the first VLC column (42 mg) was purified on ODS HPLC column (Cosmosil, 250 × 10 mm) using 45% CH_3_CN at 2 mL/min to give compounds **2** (4.1 mg) and **1** (2.7 mg). Similarly, the fraction eluted with 80% MeOH in EtOAc from the first VLC column (34 mg) was purified on ODS HPLC column (Cosmosil, 250 × 10 mm) using 65% CH_3_CN at 2 mL/min to give compounds **3** (3.7 mg) and **4** (2.8 mg).

### 3.10. Spectral Data of the Compounds

#### 3.10.1. Asperopiperazine A (**1**)

Oil; [α]_D_ +38° (c 0.1, MeOH); UV (MeOH) λ_max_ (log ε): 231 (4.06), 303 (4.15) nm; IR (film) ν_max_ 3449, 1659, 1629 cm^−1^; NMR data: see Table 1; HRESIMS *m*/*z* 339.1683 (calcd for C_18_H_24_N_2_O_3_Na [M + Na]^+^, 339.1679).

#### 3.10.2. Asperopiperazine B (**2**)

Oil; [α]_D_ −61° (c 0.1, MeOH); UV (MeOH) λ_max_ (log ε): 233 (4.03), 306 (4.17) nm; IR (film) ν_max_ 3455, 1663, 1631 cm^−1^; NMR data: see Table 1; HRESIMS *m*/*z* 283.1055 (calcd for C_14_H_16_N_2_O_3_Na [M + Na]^+^, 283.1053).

### 3.11. Absolute Stereochemistry of ***1*** and ***2***

#### 3.11.1. Preparation of l-FDLA Derivatives for HPLC Analyses

A solution of **1** and **2** (0.6 mg each) were dissolved in 6 M HCl (200 μL), separately, and heated at 110 °C overnight. After cooling and drying the mixture, 50 μL of 1% 1-fluoro-2,4-dinitrophenyl-5-l-leucinamide (l-FDLA) in acetone and 40 μL of 1.0 M NaHCO_3_ were added. The mixtures were heated at 55 °C for 30 min and were then diluted with 50% MeOH−0.2% HCO_2_H (1:1, 100 μL) before the HPLC analyses [31].

#### 3.11.2. HPLC Analysis of FDLA Derivatives

The FDLA derivatives of the hydrolysates and standards (both d and l) were analyzed by HPLC (Cadenza CD-C18, 3 μm, 2.0 × 150 mm) at 40 °C at a flow rate of 0.2 mL/min and UV absorption at 340 nm. MeCN−2% HCO_2_H was used as a mobile phase in a gradient mode (MeCN concentration: 25% for 0–4 min; 25–55% for 4–19 min; 55–100% for 19–25 min; 100% for 25–33 min). The retention times for the amino acid standards were as follows: 24.1 min (for l-Phe); 26.7 min (for d-Phe); 25.7 min (for l-NMe-Leu); 26.9 min (for d-NM-Leu), respectively (Figure S27, Supplementary Materials).

The l-FDLA-derivatized hydrolysate of **1** gave peaks at 24.1 min (similar to l-Phe) and at 25.7 min (similar to l-NMe-Leu). In addition, the l-FDLA-derivatized hydrolysate of **2** gave a peak at 26.7 min (similar to d-Phe). Thus, the amino acids were identified as l-NMe-Leu and l-Phe in asperopiperazine A (**1**) and d-Phe in asperopiperazine B (**2**).

#### 3.11.3. Computational Details

All of the DFT calculations were performed using Gaussian 16 [40]. A conformation analysis was conducted using the GMMX plugin followed by a geometry optimization at the B3LYP/6-31g(d) level. A frequency check was performed at the same level of theory. The GIAO NMR properties were calculated at the mpw1pw91/6-311+g(2d,p) level. The DP4 probabilities were calculated using our own implementation of the algorithm published by Smith and Goodman [41]. 

### 3.12. Biological Evaluation of the Compounds

#### 3.12.1. Evaluation of the Antimicrobial Effects of **1**–**4**

##### Disk Diffusion Assay

The antimicrobial effects of compounds **1**–**4** were evaluated using disc diffusion assay at 50 µg/disc against *E. coli* (ATCC 25922), *C. albicans* (ATCC 14053), and *S. aureus* (ATCC 25923), and as described before [42,43]. Ciprofloxacin and ketoconazole served as the positive controls.

##### Evaluation of the MIC Values

The determination of the MIC values of **1**–**4** was performed using a macro-dilution assay, as previously reported [44]. Briefly, The MIC values of **1**–**4** were evaluation of using a macro-dilution method [44], using MeOH to dissolve the compounds at a final concentration of 2000 μg/mL, while distilled H_2_O was used to dissolve ciprofloxacin and ketoconazole at final concentrations of 100 μg/mL. All of the solutions were sterilized using syringe filters (0.2 μm). Two-fold serial dilutions of the solutions were used in MHB to afford concentrations between 1.0 and 1000 μg/mL for the compounds, and between 0.125 and 64 μg/mL for the ciprofloxacin and ketoconazole. From the 10^6^ CFU/mL microbial suspensions, 500 μL were added in sterile tubes giving inoculua of 5 × 10^5^ CFU/mL. In addition, 100 μL of each stock solution of the compounds and antibiotics were added into the tubes. A control tube, which contained only the test microorganisms and methanol, was prepared [44]. The MeOH displayed no antimicrobial effect. Incubation of the tubes was accomplished at 37 °C for 48 h. The lowest concentrations of the compounds/antibiotics, which showed no microbial growth, were considered as MIC.

#### 3.12.2. Evaluation of the Growth Inhibition Effects of the Compounds

##### Culture of Cell Lines

The cell lines HeLa (human cervical carcinoma, ATCC CCL-2) and HCT 116 (colorectal carcinoma, ATCC CCL-247) were cultured in RPMI 1640 medium, including 1% penicillin–streptomycin and 10% FBS. The cell line MDA-MB-231 (triple-negative breast cancer, ATCC HTB-26) was cultured in DMEM medium, including 10% FBS and 1% penicillin–streptomycin.

##### MTT Assay

The evaluation of the antiproliferative and growth inhibitory effects of the compounds was carried out using MTT assay, as described before [45,46,47,48]. Briefly, the cells were incubated overnight in 5% CO_2_/air at 37 °C. The compounds were added at the top row of a 96-well microtiter plate, and descendant serial dilutions (1:4) of the concentration were carried out. After that, the cells with the compounds were incubated for 72 h. Using the Cell Titer 96 AQueous non-radioactive cell proliferation protocol, the cells’ viability was estimated at 490 nm, using a Molecular Devices Emax microplate reader.

The IC_50_ values of the compounds were evaluated using the program SoftMax^®^ PRO (https://www.moleculardevices.com/products/microplate-readers/acquisition-and-analysis-software/softmax-pro-software#gref; accessed on 1 July 2022). SoftMax^®^ Pro Software is a program that is designed to provide simple, flexible, and powerful methods for advanced data analysis. It provides analysis algorithms, ready-to-run protocols, with 21 different curve fit options. Every step is optimized for data acquired from a Molecular Devices microplate reader, or data imported from another source to simplify the analysis and reporting. Compliance tools are available for regulated laboratories providing end-to-end chain of custody (https://www.moleculardevices.com/products/microplate-readers/acquisition-and-analysis-software/softmax-pro-software#gref; accessed on 1 July 2022).

## 4. Conclusions

Chromatographic partition of the cytotoxic fractions of the tunicate-derived fungus *Aspergillus* sp. DY001 gave two new dipeptides, asperopiperazines A and B (**1** and **2**), along with (+)-citreoisocoumarin (**3**) and (−)-6,8-di-*O*-methylcitreoisocoumarin (**4**). The structures of the compounds were assigned by interpretation of their spectroscopic data, including NMR and (+)-HRESIMS. Asperopiperazines A and B inhibited both the HCT 116 and MDA-MB-231 cell lines with IC_50_ values down to 15.1 µM. On the other hand, compounds **3** and **4** were less active than **1** and **2** towards the same cells. All of the compounds were inactive towards the HeLa cells, suggesting the lack of selectivity towards this cell line. In the antimicrobial screen, **1** and **2** significantly inhibited both *E. coli* and *S. aureus* with MIC values of 8 and 4 µM (*E. coli*) and 8 and 8 µM (*S. aureus*), respectively. The results of this investigation provide a deeper insight into the diversity of the secondary metabolites of members of the genus *Aspergillus* and their biological properties.

## Figures and Tables

**Table 1:** NMR spectral data of asperopiperazines A (**1**) and B (**2**).

Position	1 (CD_3_OD) ^a^		2 (CDCl_3_) ^b^	
	δ_C_ (mult.) ^c^	δ_H_ (mult. *J* (Hz))	δ_C_ (mult.) ^c^	δ_H_ (mult. *J* (Hz))
2	174.5, C		166.1, C	
3	52.6, CH	5.00 (t, 7.8)	55.8, CH	4.45 (dd, 11.0, 3.3)
4 (N*H*)			-	5.58 (brs)
5	175.2, C		167.9, C	
6	56.3, CH	5.04 (dd, 9.6, 6.0)	87.6, C	
6-O*H*				2.98 (brs)
7	38.1, CH_2_	1.62 (m)	36.8, CH_2_	2.23 (m), 2.17 (m)
8	25.8, CH	1.42 (m)	19.9, CH_2_	2.17 (m), 2.00 (m)
9	23.6, CH_3_	0.91 (d, 6.6)	45.5, CH_2_	3.79 (m), 3.59 (m)
10	22.0, CH_3_	0.85 (d, 6.6)	36.9, CH_2_	3.63 (dd, 14.4, 4.2) 2.74 (dd, 14.4, 10.2)
11	38.7, CH_2_	3.06 (dd, 13.2, 7.6) 2.93 (dd, 13.2, 7.2)	135.7, C	
12	138.0, C		129.1, CH	7.23 (d, 7.5)
13	129.6, CH	7.25 (m)	129.3, CH	7.35 (t, 7.5)
14	130.4, CH	7.25 (m)	127.6, CH	7.29 (t, 7.5)
15	128.1, CH	7.21 (m)	129.3, CH	7.35 (t, 7.5)
16	130.4, CH	7.25 (m)	129.1, CH	7.23 (d, 7.5)
17	129.6, CH	7.25 (m)		
18	31.7, CH_3_	2.84 (s)		
19	172.8, C			
20	22.1, CH_3_	1.89 (s)		

(^a^) Recorded at 600 MHz for ^1^H and 150 MHz for ^13^C NMR; (^b^) Recorded at 850 MHz for ^1^H and 213 MHz for ^13^C NMR; (^c^) All ^13^C NMR signals were unambiguously assigned from HSQC and HMBC experiments.

**Table 2:** Comparison of the ^13^C NMR data of the 6-OH-Pro moiety in **2** and cyclo(d-6-OH-Pro-l-Phe).

Carbon No.	Compound 2	Cyclo(6-OH-d-Pro-l-Phe) [33]	Δδ_C_ (ppm)
	δ_C_ (CDCl_3_)	δ_C_ (CDCl_3_)	
C-6	87.6	87.6	0.0
C-7	36.8	36.8	0.0
C-8	19.9	19.9	0.0
C-9	45.5	45.4	−0.1

**Table 3:** Antimicrobial activities of **1**–**4**.

Compound No.	*C. albicans*		*E. coli*		*S. aureus*	
	Inhibition Zone (mm)	MIC (μM)	Inhibition Zone (mm)	MIC (μM)	Inhibition Zone (mm)	MIC (μM)
**1**	11	16	17	8	16	8
**2**	12	16	23	4	18	8
**3**	17	8	11	8	19	8
**4**	9	16	7	16	6	32
Ciprofloxacin ^a^	NT		30	0.08	22	
Ketoconazole ^b^	30	0.26	NT		NT	NT

(^a^) Positive antibacterial control (5 µg/disc); (^b^) positive antifungal control (50 µg/disc); NT = not tested.

**Table 4:** Antiproliferative activities of **1**–**4**.

Compound	IC_50_ (μM) ^a^		
	MDA-MB-231	HeLa	HCT 116
**1**	24.3 ± 0.2	≥50.0	15.1 ± 0.1
**2**	26.3 ± 0.3	≥50.0	16.2 ± 0.1
**3**	31.0 ± 0.2	≥50.0	19.3 ± 0.1
**4**	35.0 ± 0.2	≥50.0	17.7 ± 0.1
5-FU ^b^	13.0 ± 0.3	12.3 ± 0.2	4.6 ± 0.2

(^a^) The results represent the mean of three independent experiments; (^b^) 5-Flourouracil, a positive cytotoxic control.

## Data Availability

Data are contained within this article or Supplementary Material.

## Supplementary Materials

The following are available online at https://www.mdpi.com/article/10.3390/md20070451/s1, Figures S1–S27: HRESIMS, ^1^HNMR, ^13^C NMR, COSY, HSQC, HMBC and NOESY spectra of compounds **1**–**4** and HPLC chromatograms of l-derivatized standard amino acids and the hydrolysates of compounds **1** and **2**. Table S1: Calculated NMR chemical shifts of the isomers of **2**.

## References

[1] Pereira F. (2019). Have marine natural product drug discovery efforts been productive and how can we improve their efficiency?. Expert Opin. Drug Dis..

[2] Pereira F., Aires-de-Sousa J. (2018). Computational methodologies in the exploration of marine natural product leads. Mar. Drugs.

[3] Newman D.J., Cragg G.M. (2012). Natural products as sources of new drugs over the 30 years from 1981 to 2010. J. Nat. Prod..

[4] Newman D.J., Giddings L.-A. (2014). Natural products as leads to antitumor drug. Phytochem. Rev..

[5] Youssef D.T.A., Almagthali H., Shaala L.A., Schmidt E.W. (2020). Secondary metabolites of the genus *Didemnum*: A comprehensive review of chemical diversity and pharmacological properties. Mar. Drugs.

[6] Bailly C. (2015). Anticancer properties of lamellarins. Mar. Drugs.

[7] Oku N., Matsunaga S., Fusetani N. (2003). Shishijimicins A−C, novel enediyne antitumor antibiotics from the ascidian *Didemnum proliferum*. J. Am. Chem. Soc..

[8] Schmidt E.W., Donia M.S. (2010). Life in cellulose houses: Symbiotic bacterial biosynthesis of ascidian drugs and drug leads. Curr. Opin. Biotechnol..

[9] Ireland C.M., Fernandez A. (1998). Cyclic Peptide Antitumor Agent from an Ascidian. Ph.D. Thesis.

[10] Smith T.E., Pond C.D., Pierce E., Harmer Z.P., Kwan J., Zachariah M.M., Harper M.K., Wyche T.P., Matainaho T.K., Bugni T.S. (2018). Accessing chemical diversity from the uncultivated symbionts of small marine animals. Nat. Chem. Biol..

[11] Ruegger A., Kuhn M., Lichti H., Loosli H.R., Huguenin R., Quiquerez C., von Wartburg A. (1976). Cyclosporin A, a peptide metabolite from *Trichoderma polysporum* (Link ex Pers.) Rifai, with a remarkable immunosuppressive activity. Helv. Chim. Acta.

[12] Endo A., Monacolin K. (1979). A new hypocholesterolemic agent produced by a *Monascus* species. J. Antibiot..

[13] Abraham E.P., Newton G.G., Crawford K., Burton H.S., Hale C.W. (1953). Cephalosporin N: A new type of penicillin. Nature.

[14] Nesbitt B.F., O’Kelly J., Sargeant K., Sheridan A. (1962). *Aspergillus flavus* and turkey X disease. Toxic metabolites of *Aspergillus flavus*. Nature.

[15] Flieger M., Wurst M., Shelby R. (1997). Ergot alkaloids sources, structures, and analytical methods. Folia Microbiol..

[16] Freeman G.G., Gill J.E. (1950). Alkaline hydrolysis of trichothecin. Nature.

[17] Sun W., Wu W., Liu X., Zaleta-Pinet D.A., Clark B.R. (2019). Bioactive compounds isolated from marine-derived microbes in China: 2009–2018. Mar. Drugs.

[18] Liu L., Zheng Y.-Y., Shao C.-L., Wang C.-Y. (2019). Metabolites from marine invertebrates and their symbiotic microorganisms: Molecular diversity discovery, mining, and application. Mar. Life Sci. Technol..

[19] Wang C., Tang S., Cao S. (2021). Antimicrobial compounds from marine fungi. Phytochem. Rev..

[20] Wu B., Wiese J., Labes A., Kramer A., Schmaljohann R., Imhoff J.F. (2015). Lindgomycin, an unusual antibiotic polyketide from a marine fungus of the Lindgomycetaceae. Mar. Drugs.

[21] Xu L., Meng W., Cao C., Wang J., Shan W., Wang Q. (2015). Antibacterial and antifungal compounds from marine fungi. Mar. Drugs.

[22] Orfali R., Aboseada M.A., Abdel-Wahab N.M., Hassan H.M., Perveen S., Ameen F., Abdelmohsen U.R. (2021). Recent updates on the bioactive compounds of the marine-derived genus *Aspergillus*. RSC Adv..

[23] Wang Y., Zhang H., Gigant B., Yu Y., Wu Y., Chen X., Lai Q., Yang Z., Chen Q., Yang J. (2016). Structures of a diverse set of colchicine binding site inhibitors in complex with tubulin provide a rationale for drug discovery. FEBS J..

[24] Prota A.E., Danel F., Bachmann F., Bargsten K., Buey R.M., Pohlmann J., Reinelt S., Lane H., Steinmetz M.O. (2014). The novel microtubule-destabilizing drug BAL27862 binds to the colchicine site of tubulin with distinct effects on microtubule organization. J. Mol. Biol..

[25] Blayney D.W., Zhang Q., Feng J., Zhao Y., Bondarenko I., Vynnychenko I., Kovalenko N., Nair S., Ibrahim E., Udovista D.P. (2020). Efficacy of Plinabulin vs Pegfilgrastim for prevention of chemotherapy-induced neutropenia in adults with non–small cell lung cancer. A Phase 2 Randomized Clinical Trial. JAMA Oncol..

[26] Huggett B. (2019). Innovation’ nation. Nat. Biotechnol..

[27] Shaala L.A., Alzughaibi T., Genta-Jouve G., Youssef D.T.A. (2021). Fusaripyridines A and B; Highly oxygenated antimicrobial alkaloid dimers featuring an unprecedented 1,4-bis(2-hydroxy-1,2-dihydropyridin-2-yl)butane-2,3-dione core from the marine fungus *Fusarium* sp. LY019. Mar. Drugs.

[28] Watanabe A., Ono Y., Fujii I., Sankawa U., Mayorga M.E., Timberlake W.E., Ebizuka Y. (1998). Product identification of polyketide synthase coded by *Aspergillus nidulans wA* gene. Tetrahedron Lett..

[29] Lai S., Shizuri Y., Yamamura S., Kawai K., Furukawa H. (1991). Three new phenolic metabolites from *Penicillium* species. Heterocycles.

[30] Sun K., Li Y., Guo L., Wang Y., Liu P., Zhu W. (2014). Indole diterpenoids and isocoumarin from the fungus, *Aspergillus flavus*, isolated from the Prawn, *Penaeus vannamei*. Mar. Drugs.

[31] Shaala L.A., Youssef D.T.A., Badr J.M., Harakeh S.M., Genta-Jouve G. (2019). Bioactive diketopiperazines and nucleoside derivatives from a sponge-derived *Streptomyces* species. Mar. Drugs.

[32] Marfey P. (1984). Determination of D-amino acids. II. Use of a bifunctional reagent, 1,5-difluoro-2,4-dinitrobenzene. Carlsberg Res. Commun..

[33] Park Y.C., Gunasekera S.P., Lopez J.V., McCarthy P.J., Wright A.E. (2006). Metabolites from the marine-derived fungus *Chromocleista* sp. isolated from a deep-water sediment sample collected in the Gulf of Mexico. J. Nat. Prod..

[34] Shaala L.A., Youssef D.T.A. (2015). Identification and bioactivity of compounds from the fungus *Penicillium* sp. CYE-87 isolated from a marine tunicate. Mar. Drugs.

[35] White T.J., Bruns T., Lee S., Taylor J., Innis M.A., Gelfand D.H., Sninsky J.J., White T.J. (1990). Amplification and Direct Sequencing of Fungal Ribosomal RNA Genes for Phylogenetics. PCR Protocols: A Guide to Methods and Application.

[36] National Center for Biotechnology Information. http://www.ncbi.nlm.nih.gov.

[37] Thompson J.D., Gibson T.J., Plewniak F., Jeanmougin F., Higgins D.G. (1997). The ClustalX windows interface: Flexible strategies for multiple sequence alignment aided by quality analysis tools. Nucleic Acids Res..

[38] Hall T.A. (1999). BioEdit: A user friendly biological sequence alignment editor and analysis program for Windows 95/98/NT. Nucleic Acids Symp. Ser..

[39] Tamura K., Peterson D., Peterson N., Stecher G., Nei M., Kumar S. (2011). MEGA5: Molecular evolutionary genetics analysis using maximum likelihood, evolutionary distance, and maximum parsimony methods. Mol. Biol. Evol..

[40] Frisch M.J., Trucks G.W., Schlegel H.B., Scuseria G.E., Robb M.A., Cheeseman J.R., Scalmani G., Barone V., Mennucci B., Petersson G.A. (2009). G09a: Gaussian 09.

[41] Smith S.G., Goodman J.M. (2010). Assigning stereochemistry to single diastereoisomers by GIAO NMR calculation:The DP4 Probability. J. Am. Chem. Soc..

[42] Acar J.F., Lorian V. (1980). The disc susceptibility test. Antibiotics in Laboratory Medicine.

[43] Kiehlbauch J.A., Hannett G.E., Salfinger M., Archinal W., Monserrat C., Carlyn C. (2000). Use of the National Committee for Clinical Laboratory Standards Guidelines for Disk Diffusion Susceptibility Testing in New York State Laboratories. J. Clin. Microbiol..

[44] CLSI, Clinical and Laboratory Standards Institute (2007). Performance Standards for Antimicrobial Disk Susceptibility Tests.

[45] Youssef D.T.A., Shaala L.A., Altyar A.E. (2022). Cytotoxic phenylpropanoid derivatives and alkaloids from the flowers of *Pancratium maritimum* L.. Plants.

[46] Shaala L.A., Youssef D.T.A. (2019). Cytotoxic psammaplysin analogues from the Verongid Red Sea sponge *Aplysinella* species. Biomolecules.

[47] Youssef D.T.A., Mooberry S.L. (2006). Hurghadolide A and swinholide I, potent actin-microfilament disrupters from the Red Sea sponge *Theonella swinhoei*. J. Nat. Prod..

[48] Shaala L.A., Youssef D.T.A. (2021). Hemimycalins, C.-E: Cytotoxic and antimicrobial alkaloids with hydantoin and 2-iminoimidazolidin4-one backbones from the Red Sea marine sponge *Hemimycale* sp.. Mar. Drugs.

