# Supplementary material for: Asperopiperazines A and B: Antimicrobial and Cytotoxic Dipeptides from a Tunicate-Derived Fungus Aspergillus sp. DY001
Source: Mar Drugs. 2022 Jul 10;20(7):451. doi: 10.3390/md20070451 (PMC9319533; doi:10.3390/md20070451)

Figure S1. HRESIMS spectrum of asperopiperazine A (**1**) 1
Figure S2. 600 MHz ^{1}H NMR spectrum of asperopiperazine A (**1**) (CD_3OD) 2
Figure S3. 150 MHz ^{13}C NMR spectrum of asperopiperazine A (**1**) (CD_3OD) 4
Figure S4. DEPT spectrum of asperopiperazine A (**1**) (CD_3OD) 5
Figure S5. COSY spectrum of asperopiperazine A (**1**) (CD_3OD) 6
Figure S6. HSQC spectrum of asperopiperazine A (**1**) (CD_3OD) 7
Figure S7. HMBC spectrum of asperopiperazine A (**1**) (CD_3OD) 8
Figure S8. HRESIMS spectrum of asperopiperazine B (**2**) 9
Figure S9. 850 MHz ^{1}H NMR spectrum of asperopiperazine B (**2**) ($CDCl_3$) 10
Figure S10. 213 MHz ^{13}C NMR spectrum of asperopiperazine B (**2**) ($CDCl_3$) 12
Figure 11. COSY spectrum of asperopiperazine B (**2**) ($CDCl_3$) 13
Figure S12. HSQC spectrum of asperopiperazine B (**2**) ($CDCl_3$) 14
Figure S13. HMBC spectrum of asperopiperazine B (**2**) ($CDCl_3$) 15
Figure S14. NOESY spectrum of asperopiperazine B (**2**) ($CDCl_3$) 16
Figure S15. HRESIMS spectrum of (+)-citreoisocoumarin (**3**) 17
Figure S16. 600 MHz ^{1}H NMR spectrum of (+)-citreoisocoumarin (**3**) ($CDCl_3$) 18
Figure S17. 150 MHz ^{1}H NMR spectrum of (+)-citreoisocoumarin (**3**) ($CDCl_3$) 20
Figure S18. COSY spectrum of (+)-citreoisocoumarin (**3**) ($CDCl_3$) 21
Figure S19. HSQC NMR spectrum of (+)-citreoisocoumarin (**3**) ($CDCl_3$) 22
Figure S20. HMBC NMR spectrum of (+)-citreoisocoumarin (**3**) ($CDCl_3$) 23
Figure S21. HRESIMS spectrum of (−)-6,8-di-*O*-methylcitreoisocoumarin (**4**) 24
Figure S22. 600 MHz ^{1}H NMR spectrum of (−)-6,8-di-*O*-methylcitreoisocoumarin (**4**) ($CDCl_3$) 25
Figure S23. 150 MHz ^{13}C NMR spectrum of (−)-6,8-di-*O*-methylcitreoisocoumarin (**4**) ($CDCl_3$) 27
Figure S24. COSY spectrum of (−)-6,8-di-*O*-methylcitreoisocoumarin (**4**) ($CDCl_3$) 28
Figure S25. HSQC spectrum of (−)-6,8-di-*O*-methylcitreoisocoumarin (**4**) ($CDCl_3$) 29
Figure S26. HMBC spectrum of (−)-6,8-di-*O*-methylcitreoisocoumarin (**4**) ($CDCl_3$) 30
Figure 27. HPLC chromatograms of L-FDLA derivatized standard amino acid and the hydrolysates of compounds **1** and **2** 31

Figure S1. HRESIMS spectrum of asperopiperazine A (**1**).

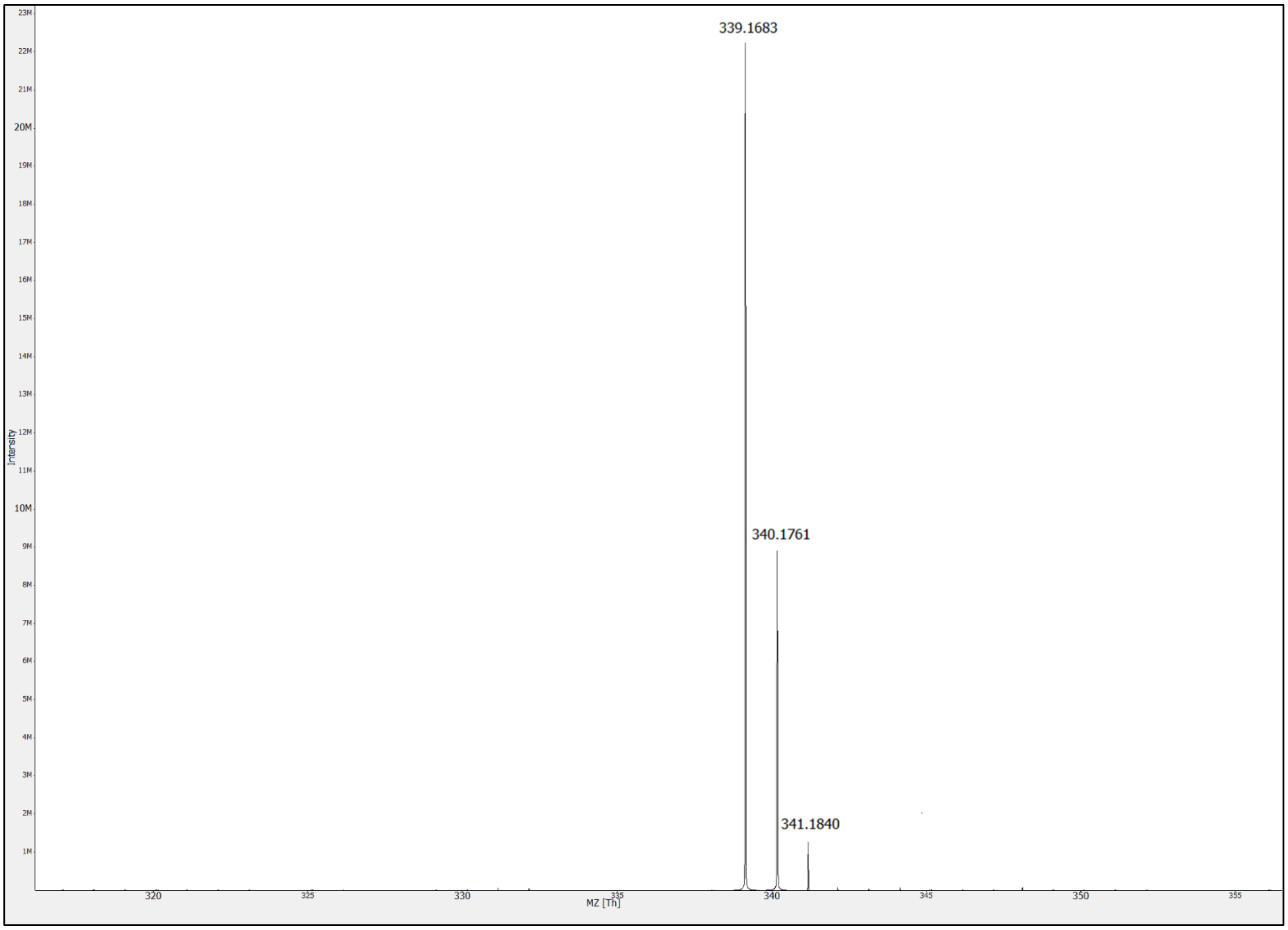

Figure S2. 600 MHz ^{1}H NMR spectrum of asperopiperazine A (**1**) (CD_3OD).

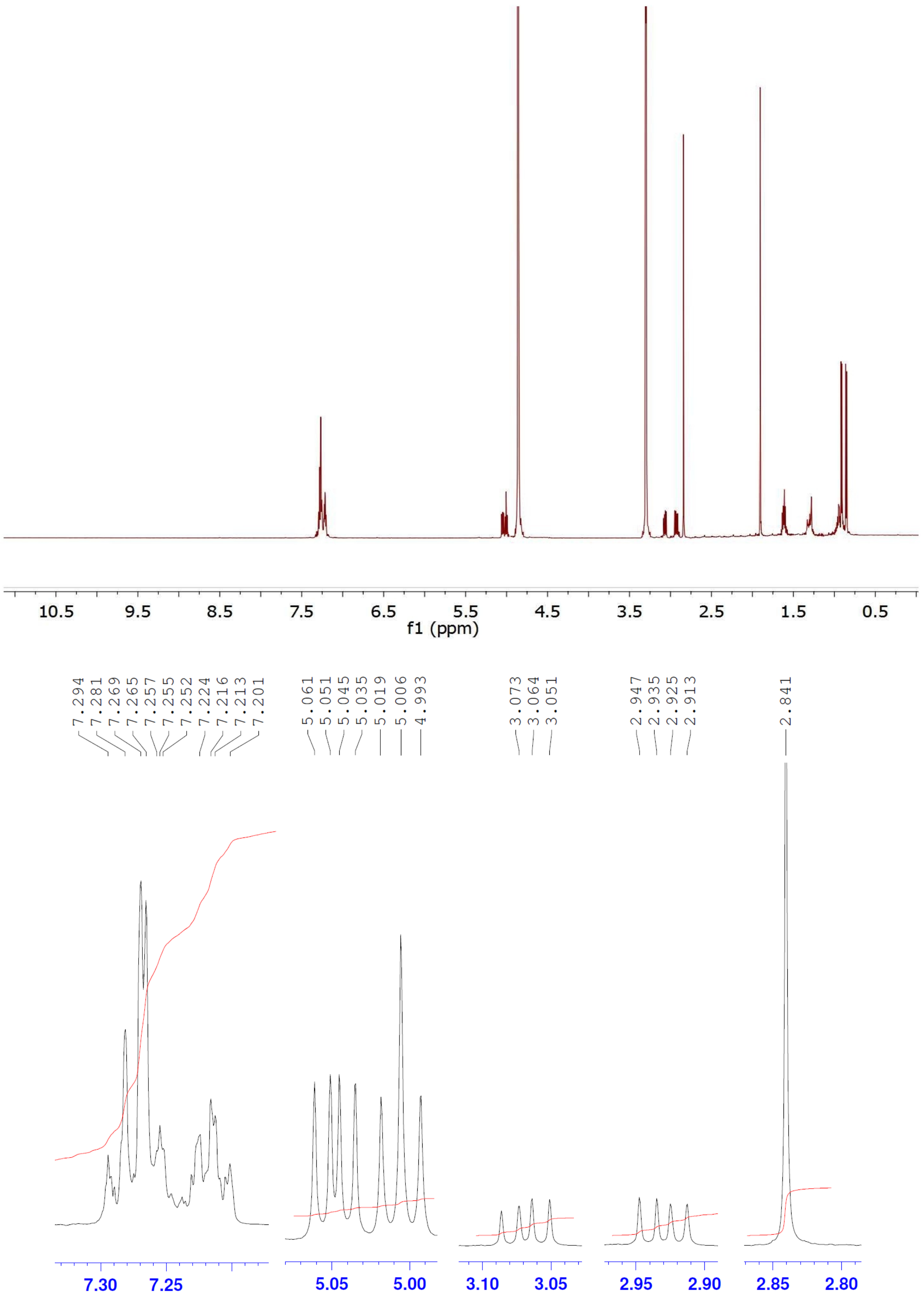

Figure S2. 600 MHz ^{1}H NMR spectrum of asperopiperazine A (**1**) (CD_3OD) (Cont.).

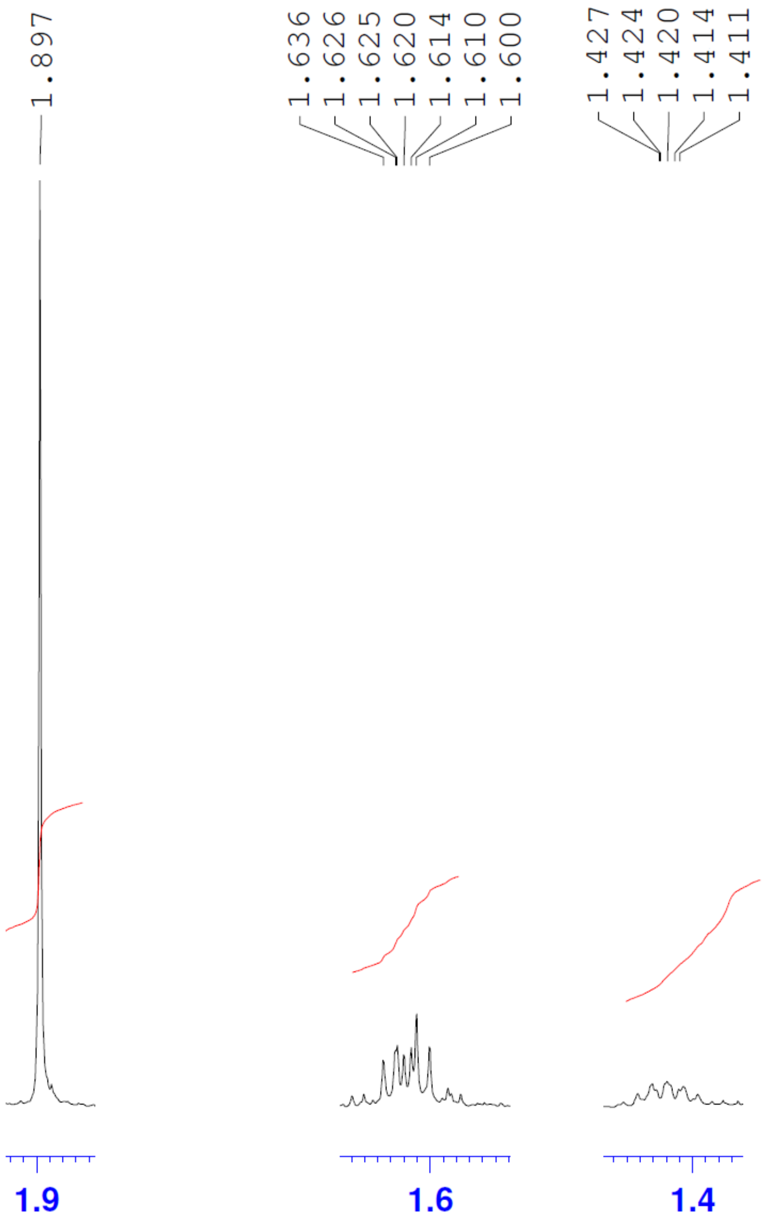

Figure S3. 150 MHz ^{13}C NMR spectrum of asperopiperazine A (**1**) (CD_3OD).

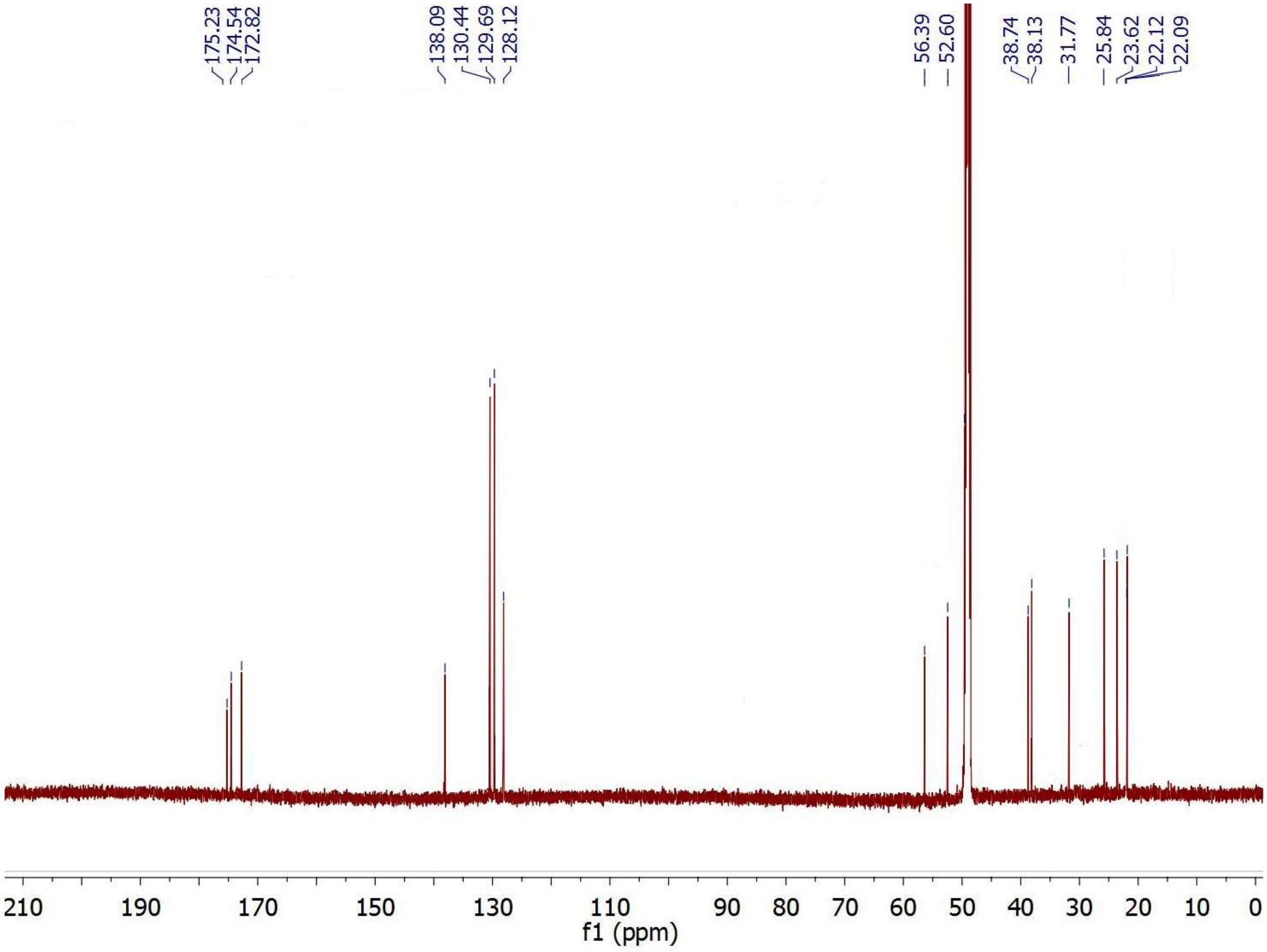

Figure S4. DEPT spectrum of asperopiperazine A (**1**) (CD_3OD).

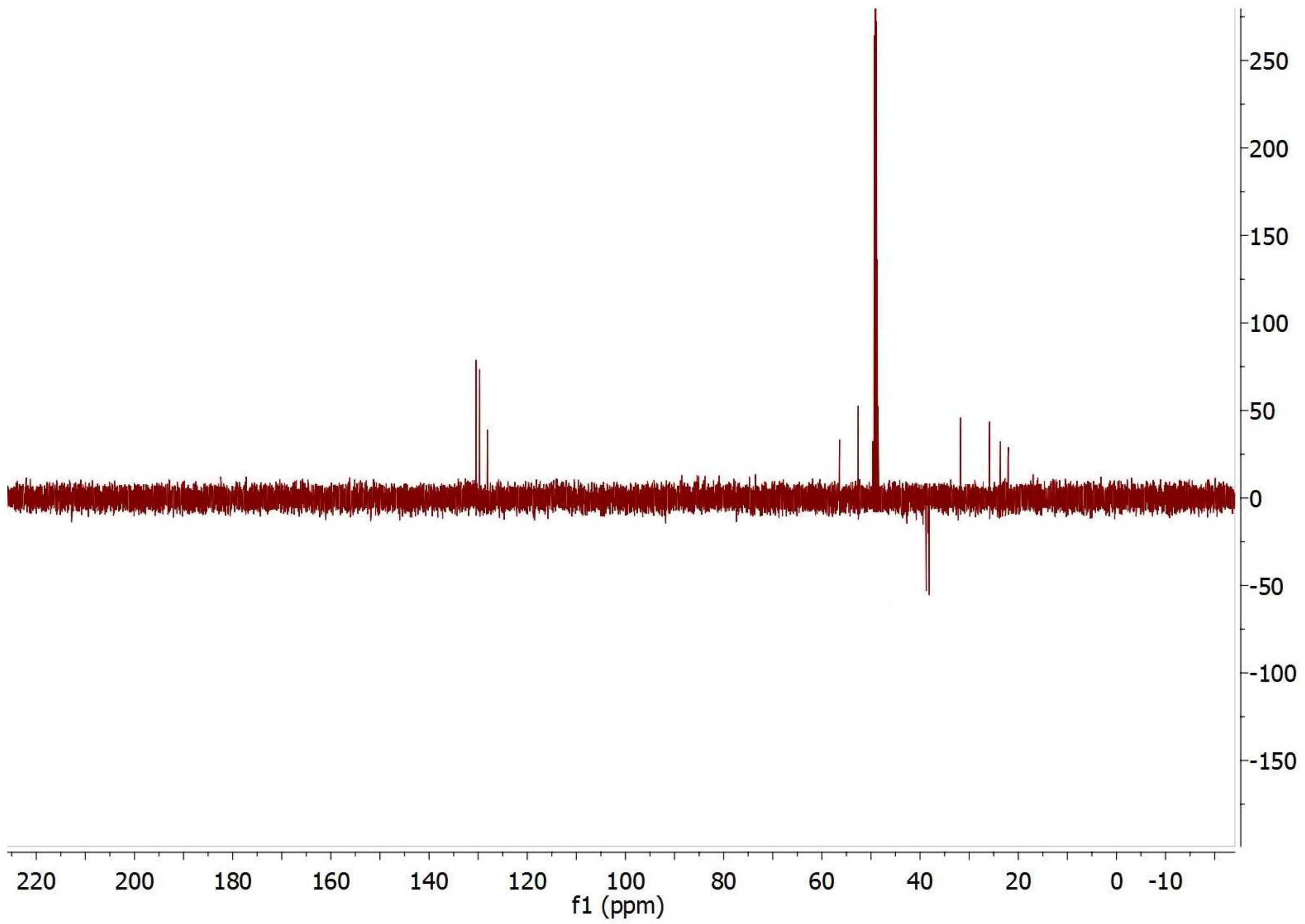

Figure S5. COSY spectrum of asperopiperazine A (**1**) (CD_3OD).

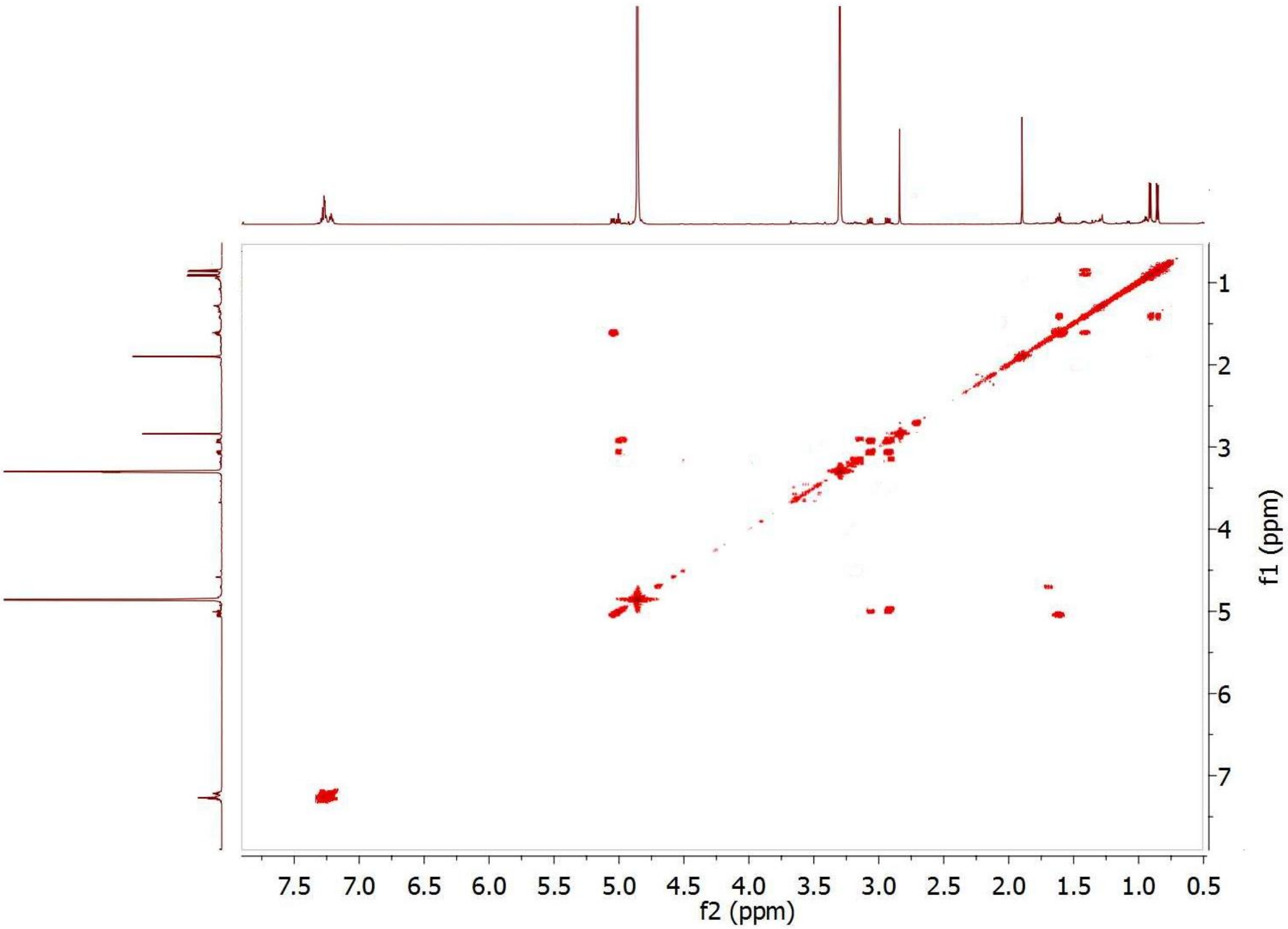

Figure S6. HSQC spectrum of asperopiperazine A (**1**) (CD_3OD).

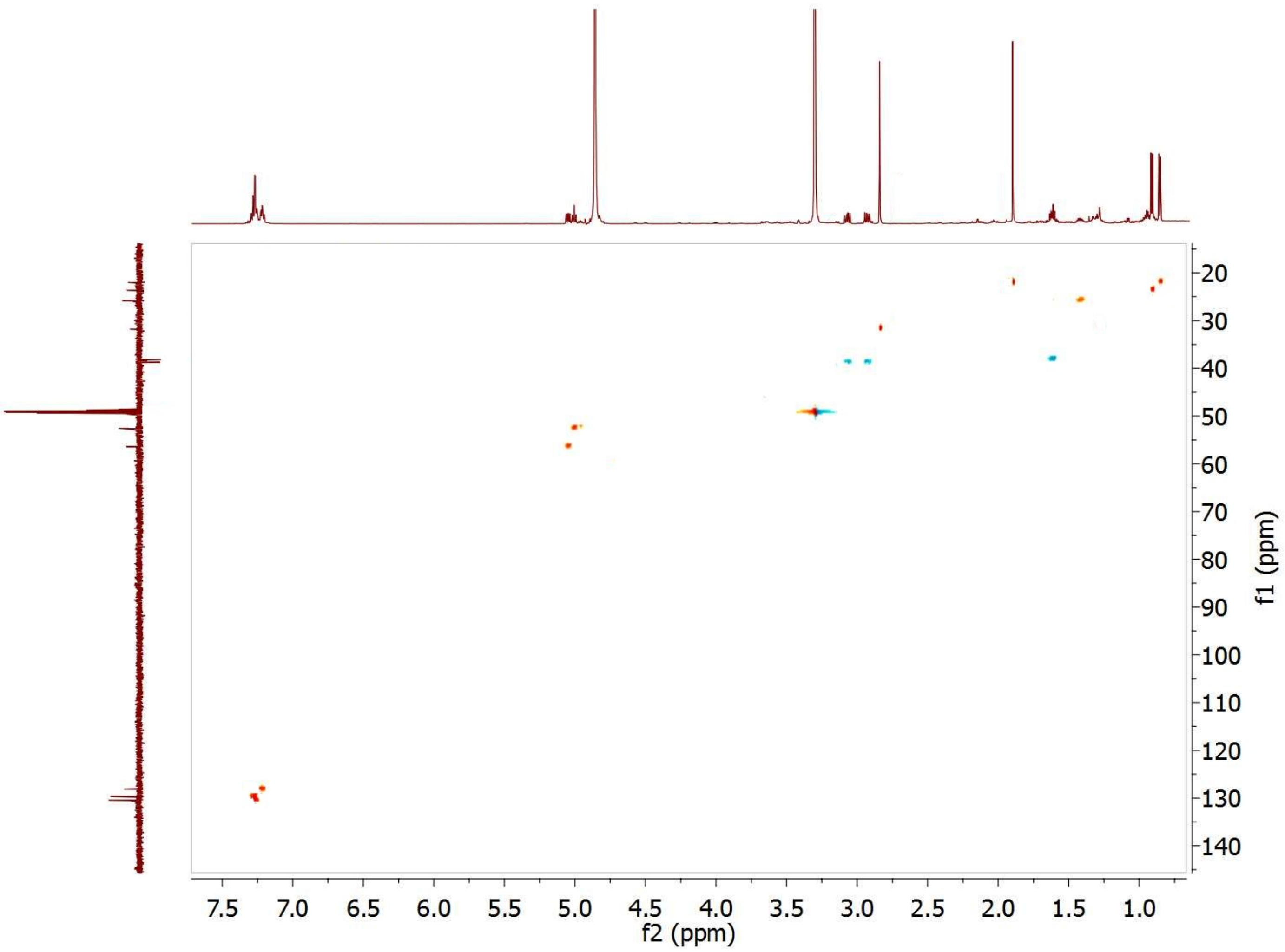

Figure S7. HMBC spectrum of asperopiperazine A (**1**) (CD_3OD).

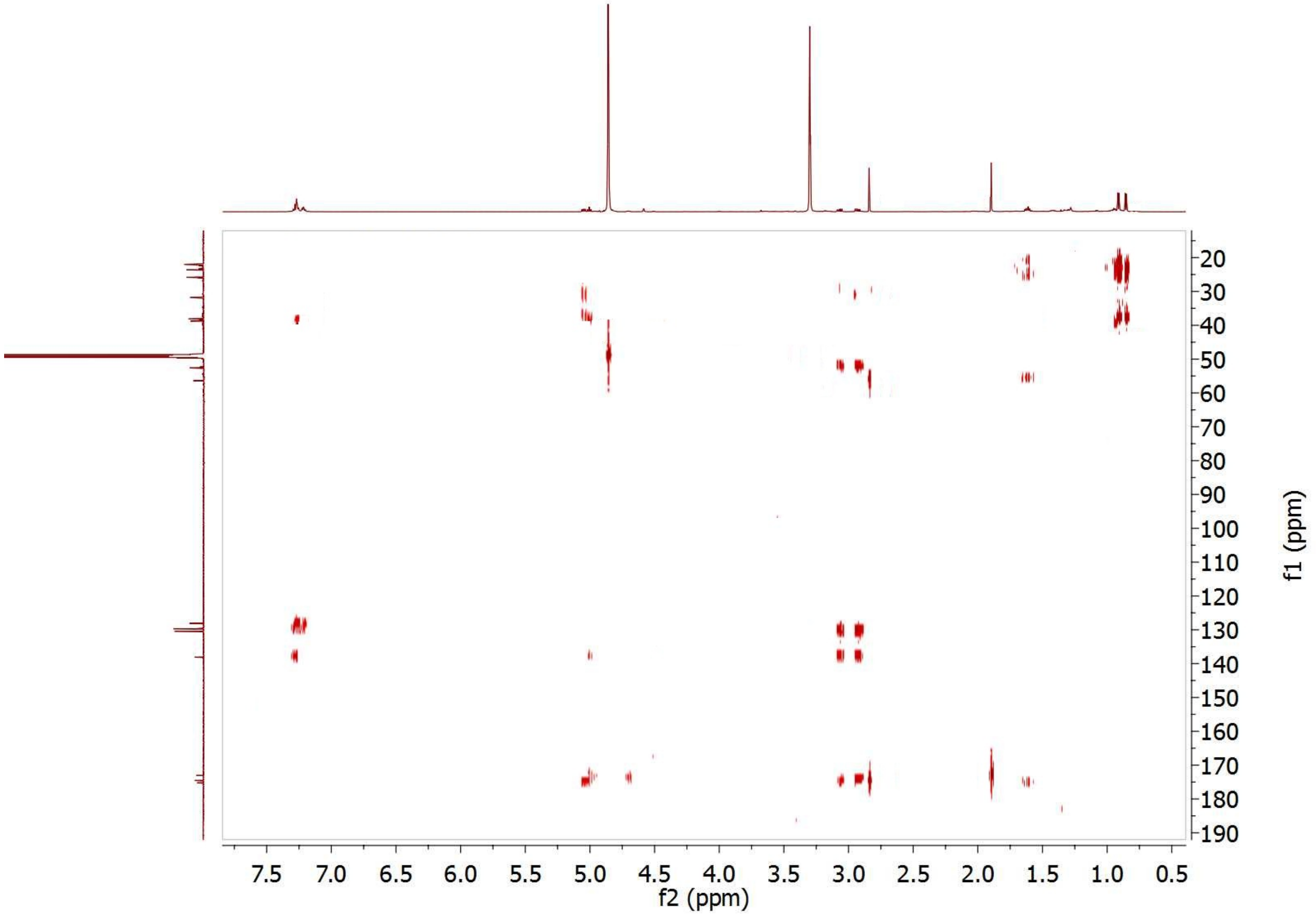

Figure S8. HRESIMS spectrum of asperopiperazine B (**2**).

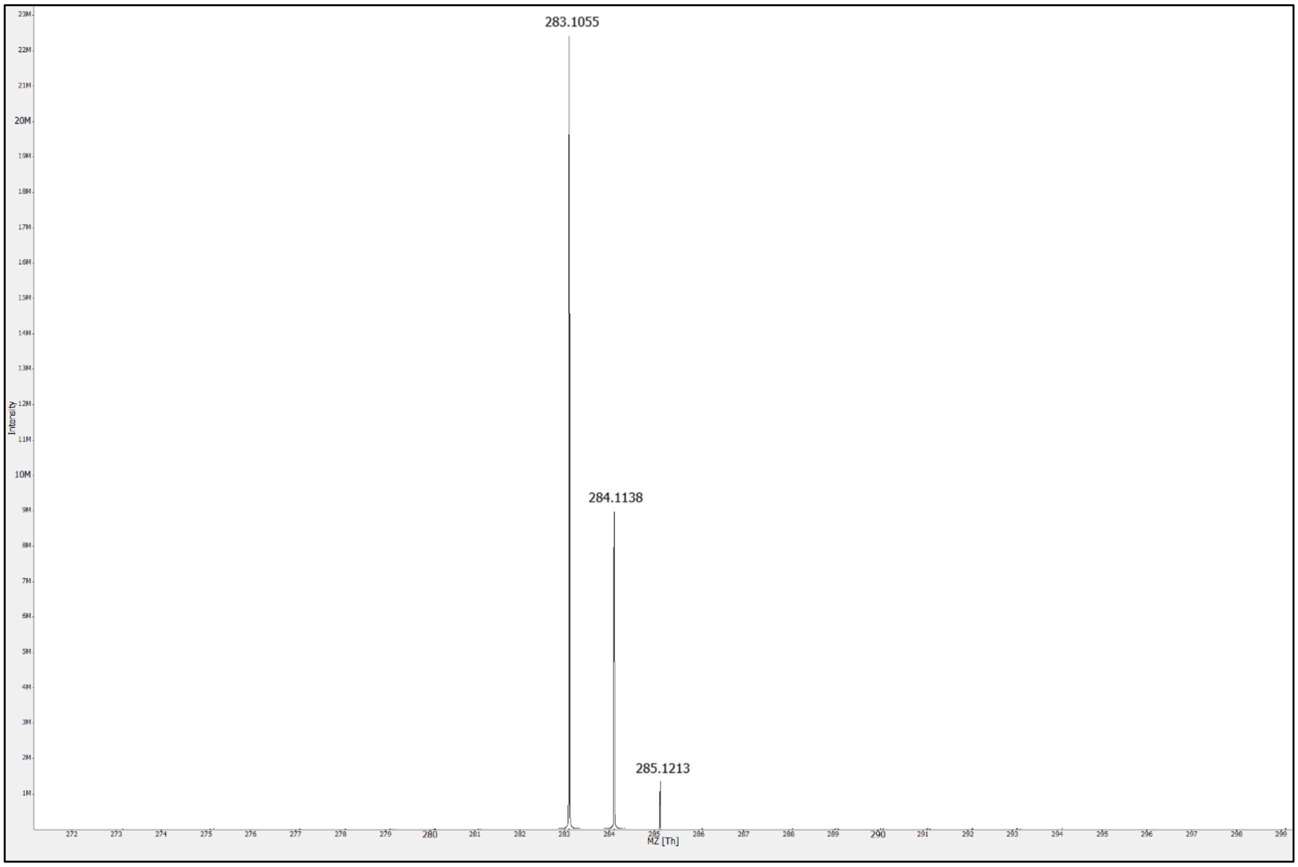

Figure S9. 850 MHz ^{1}H NMR spectrum of asperopiperazine B (**2**) ($CDCl_3$).

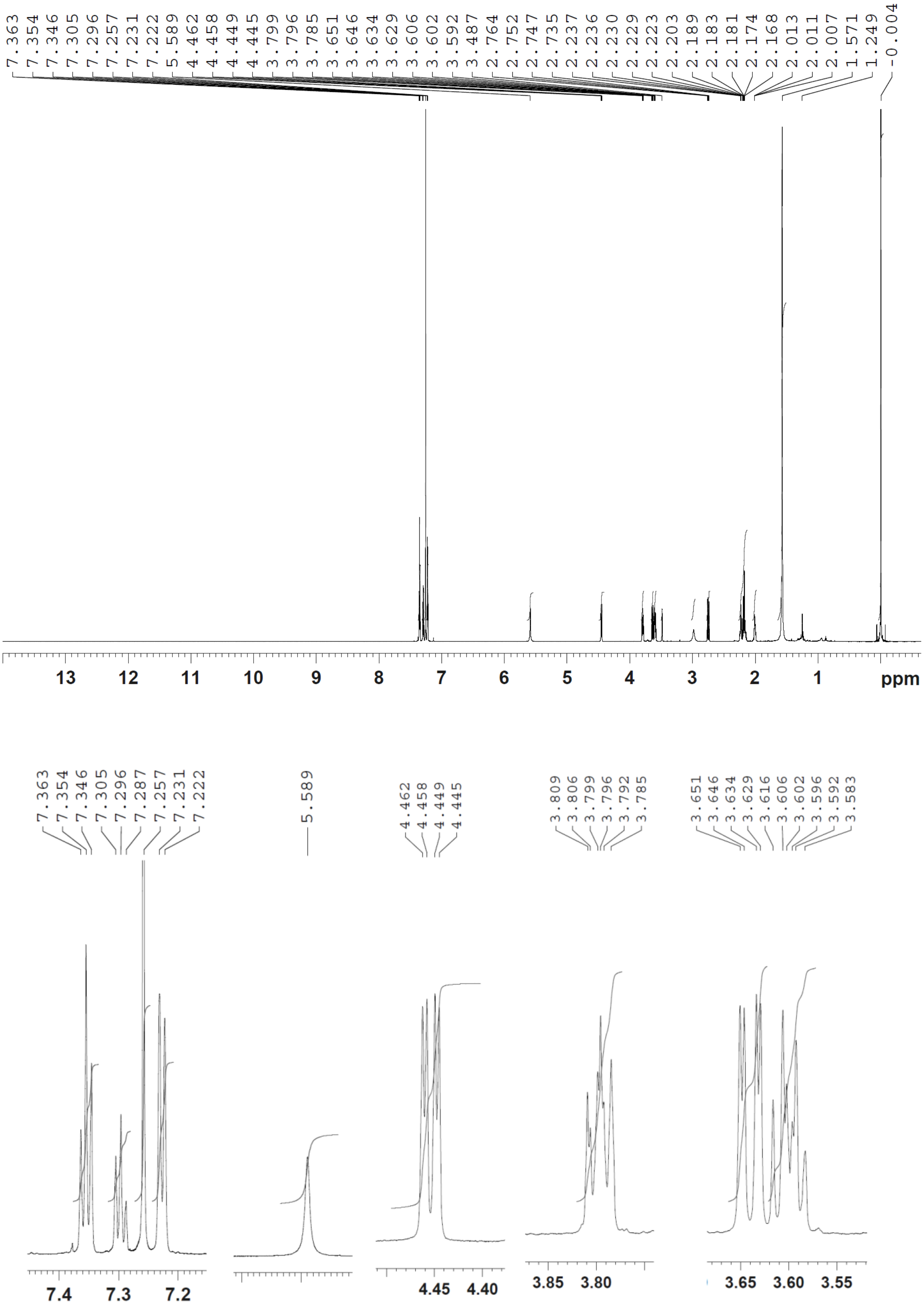

Figure S9. 850 MHz ^{1}H NMR spectrum of asperopiperazine B (**2**) ($CDCl_3$) (Cont.).

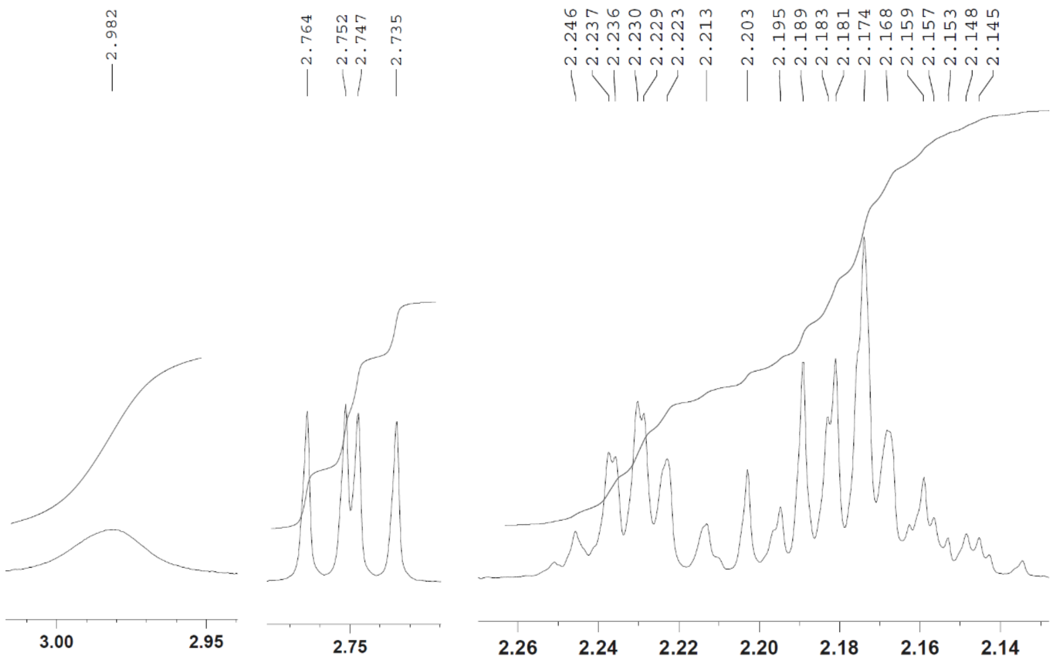

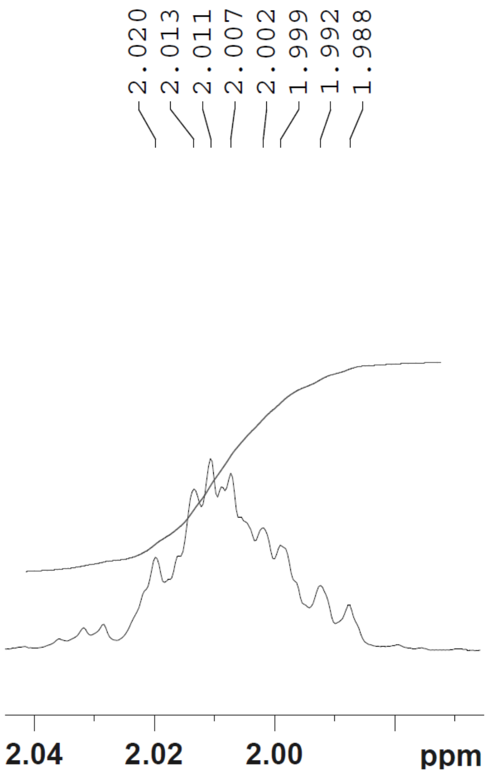

Figure S10. 213 MHz ^{13}C NMR spectrum of asperopiperazine B (**2**) ($CDCl_3$).

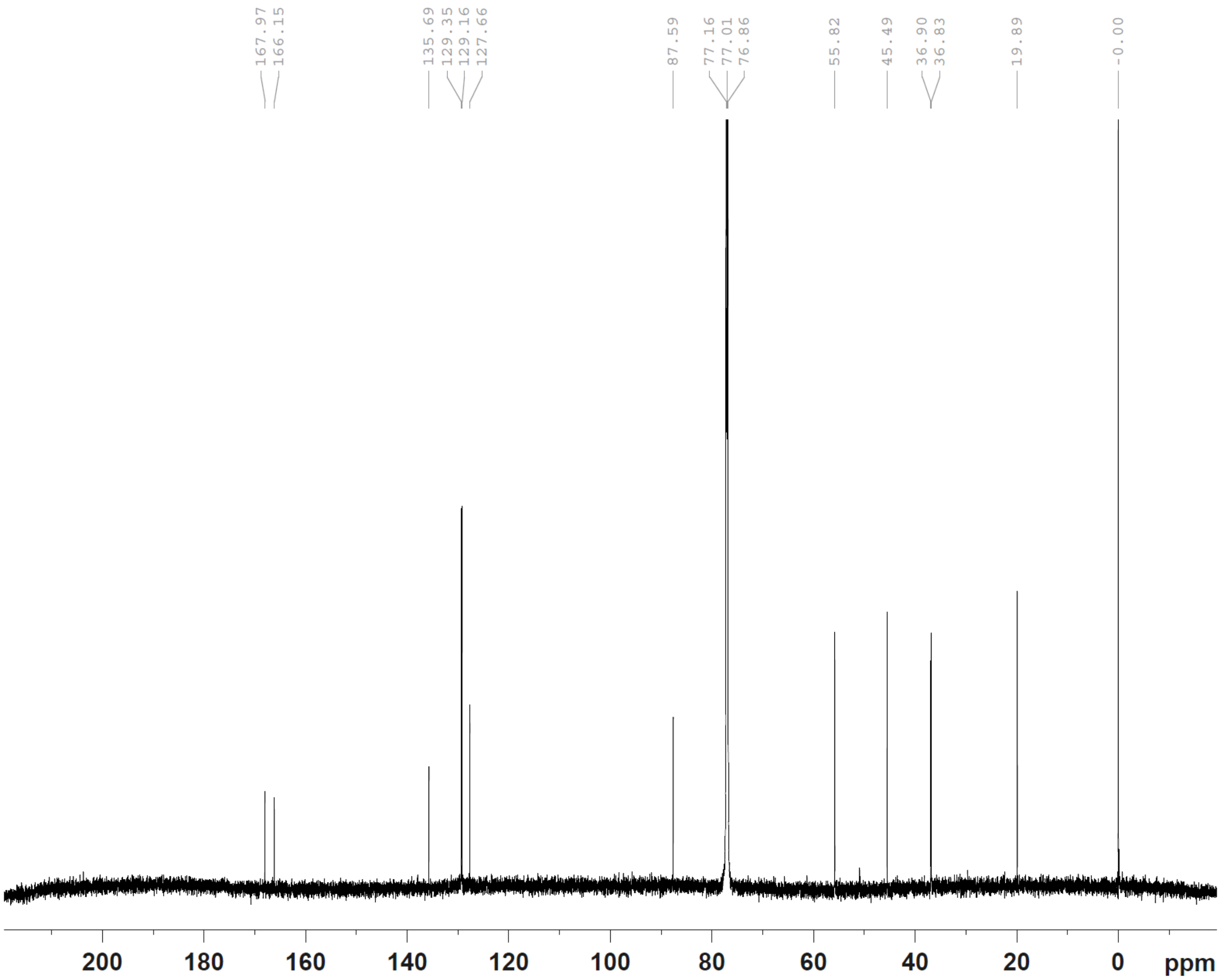

Figure S11. COSY spectrum of asperopiperazine B (**2**) ($CDCl_3$).

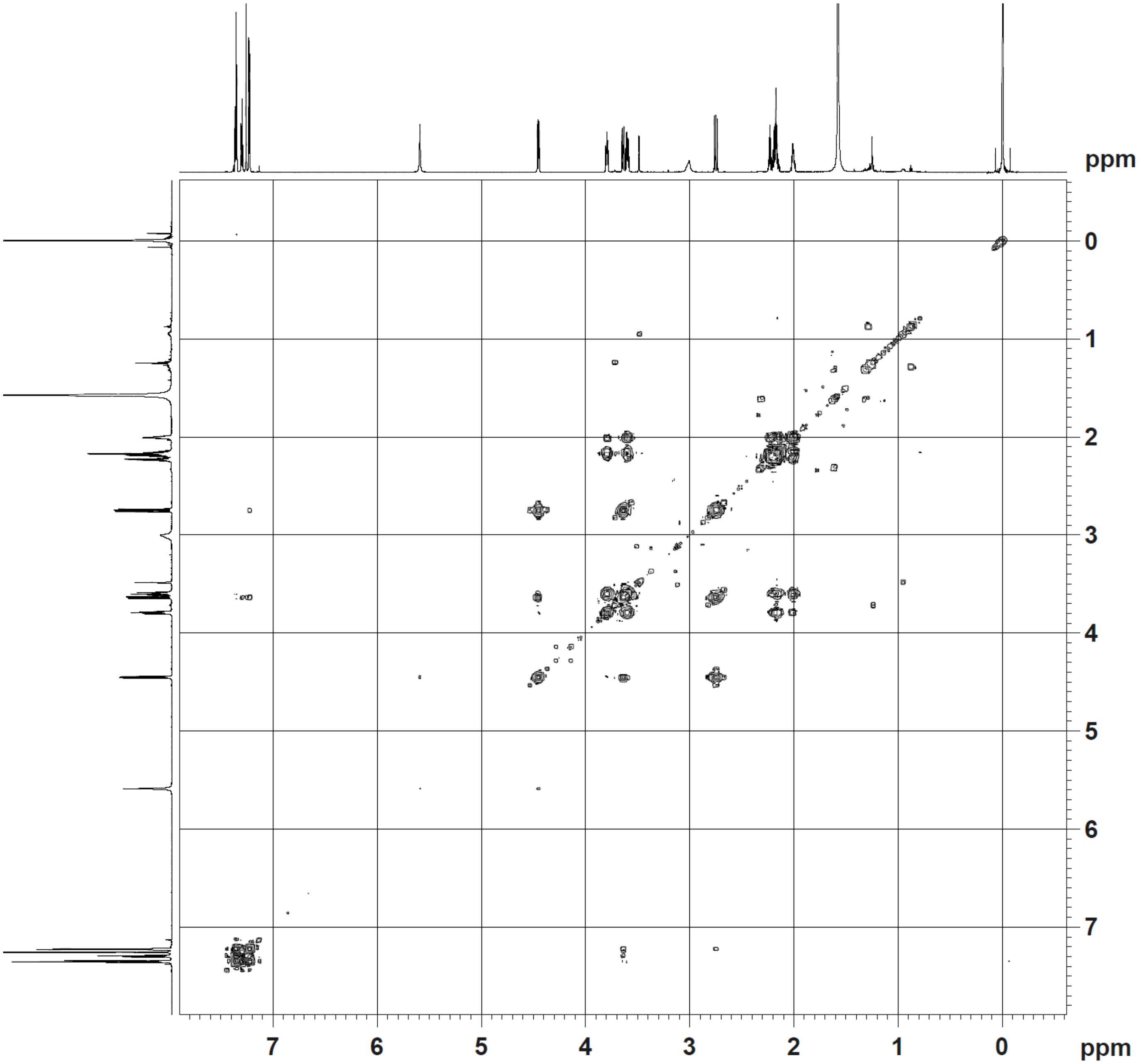

Figure S12. HSQC spectrum of asperopiperazine B (**2**) ($CDCl_3$).

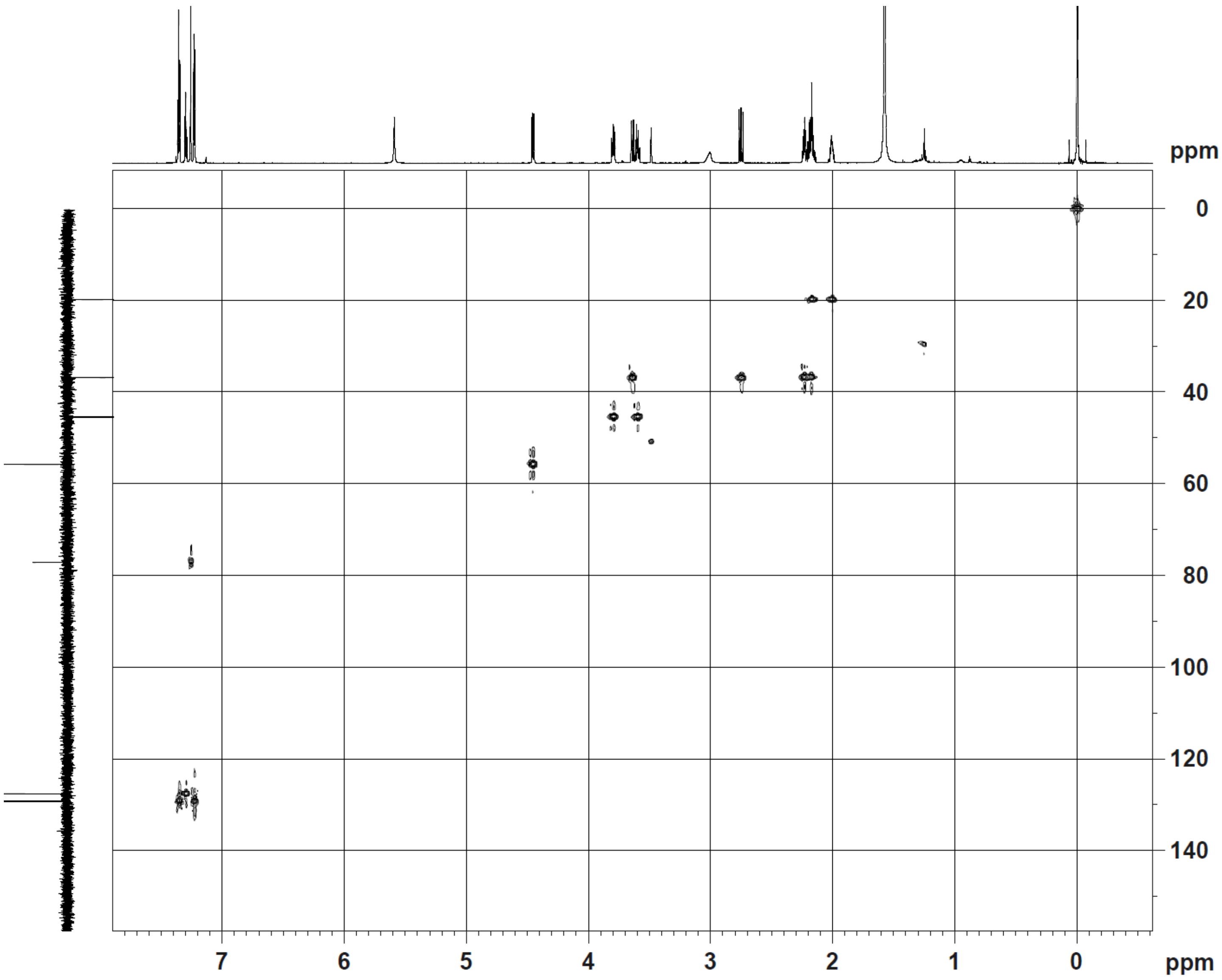

Figure S13. HMBC spectrum of asperopiperazine B (**2**) ($CDCl_3$).

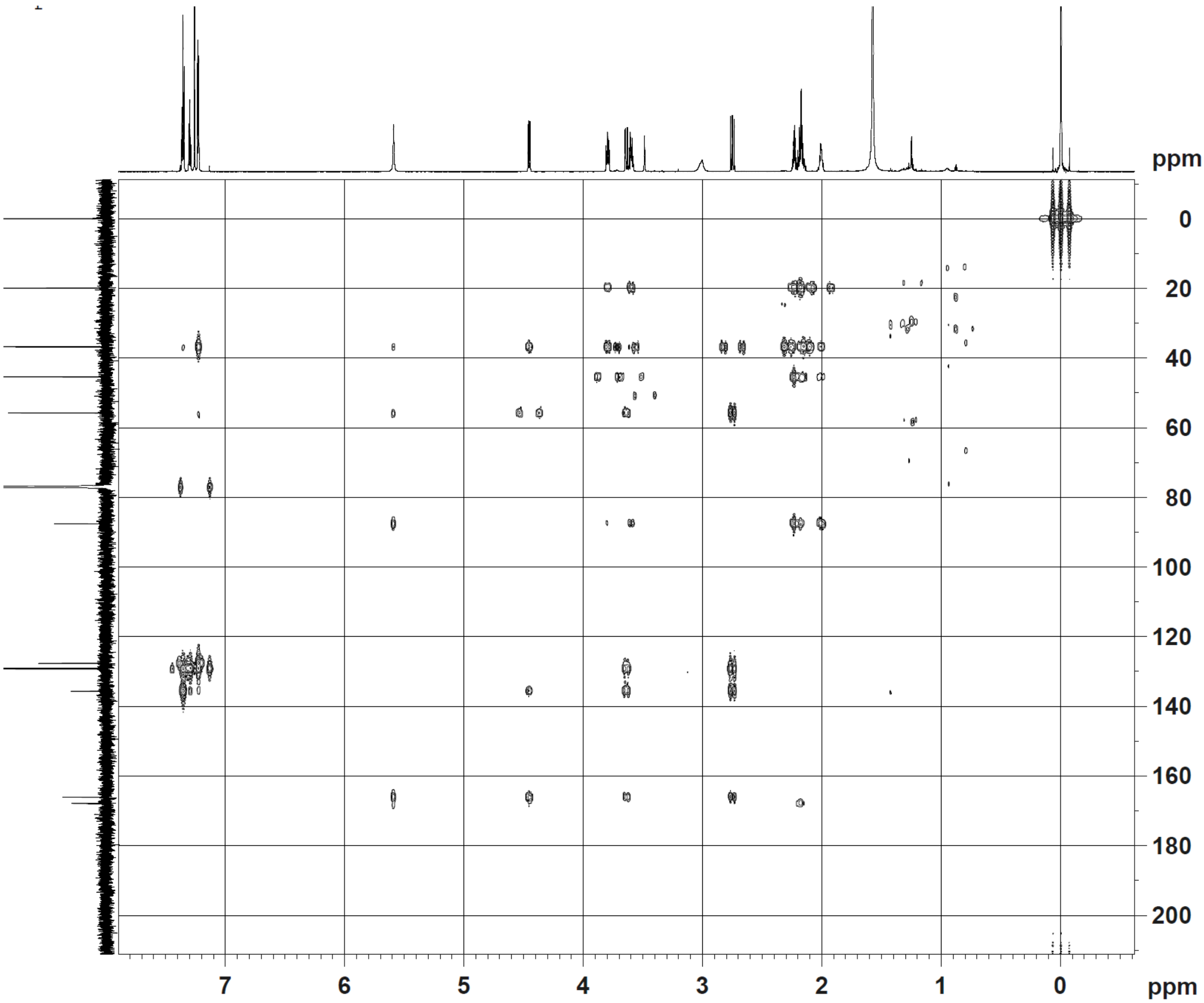

Figure S14. NOESY spectrum of asperopiperazine B (**2**) ($CDCl_3$).

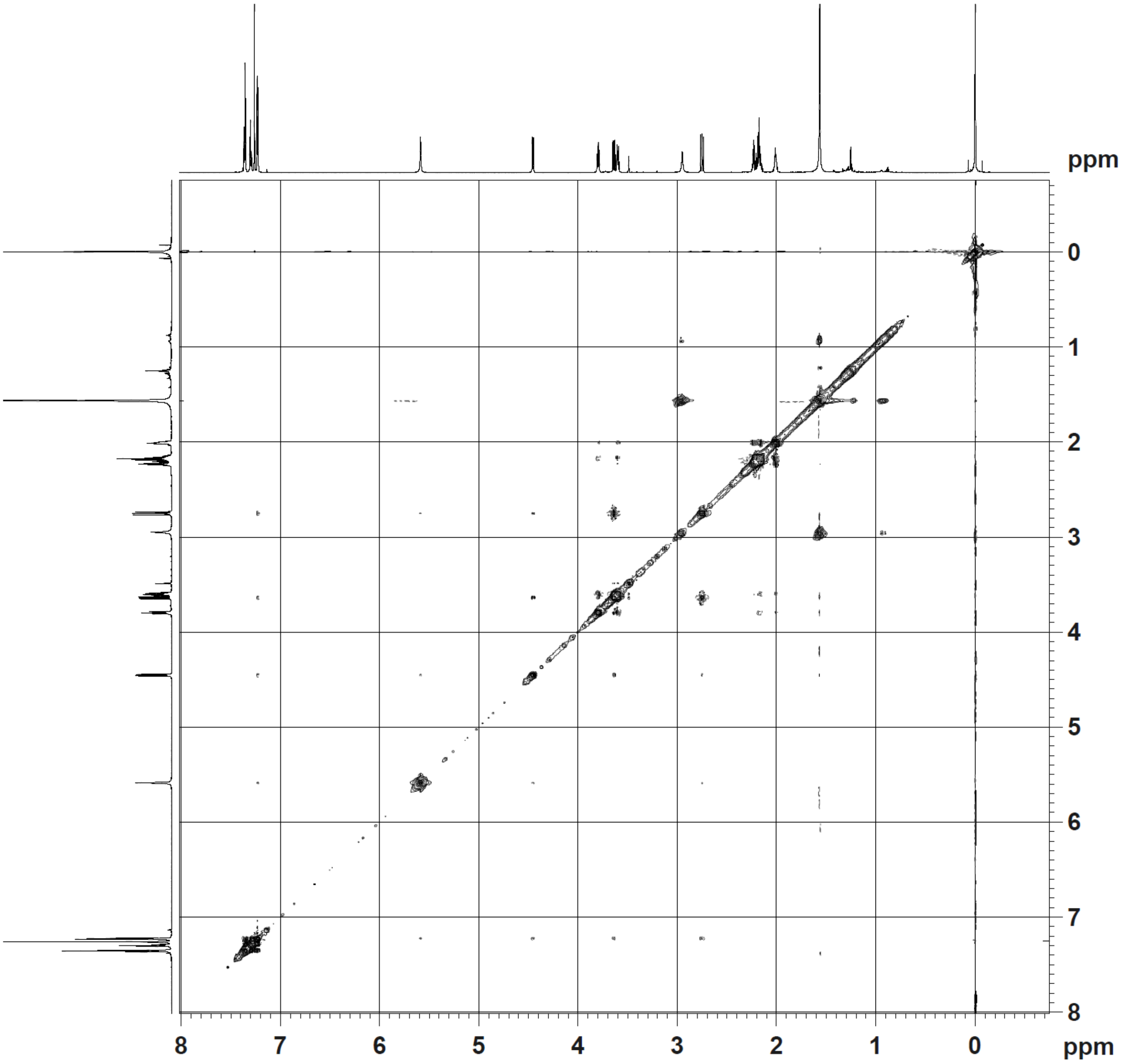

Figure 15. HRESIMS spectrum of (+)-citreoisocoumarin (**3**).

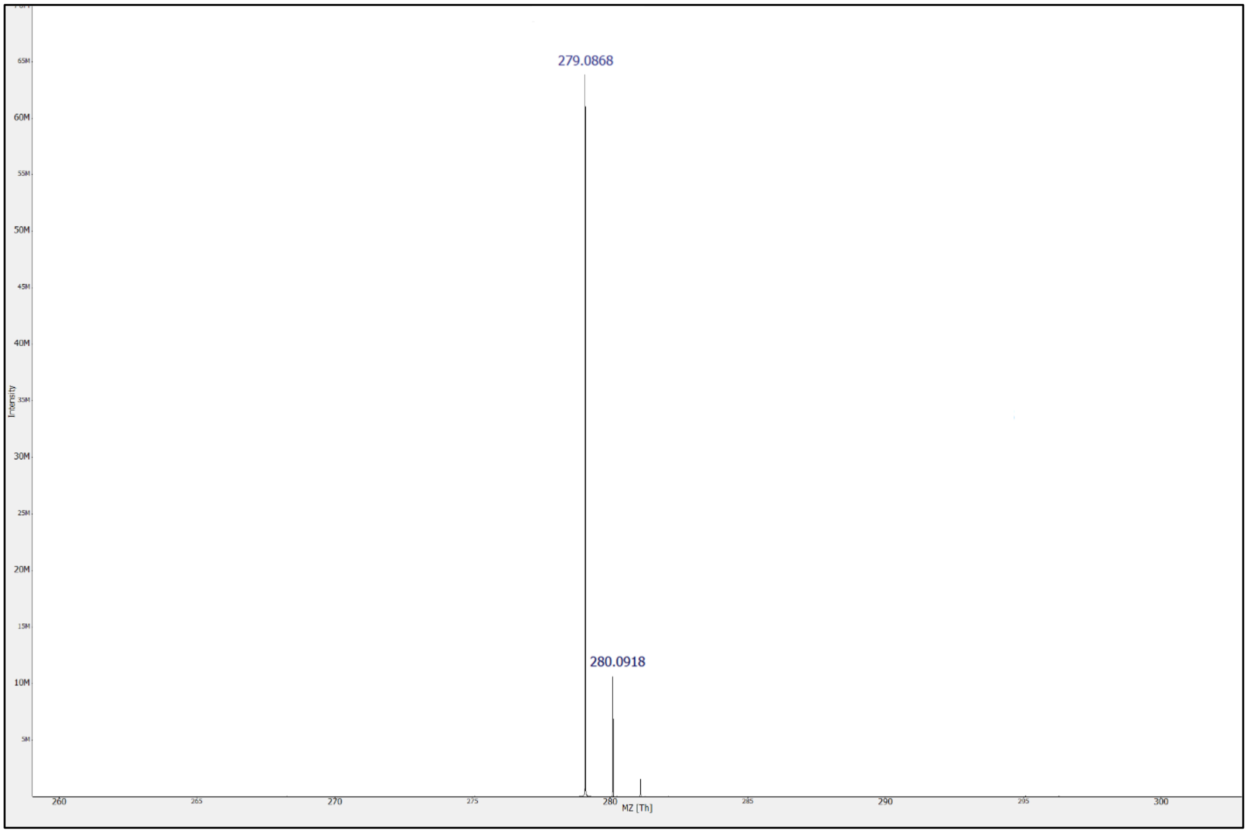

Figure S16. 600 MHz ¹H NMR spectrum of (+)-citreoisocoumarin (**3**) ($CDCl_3$).

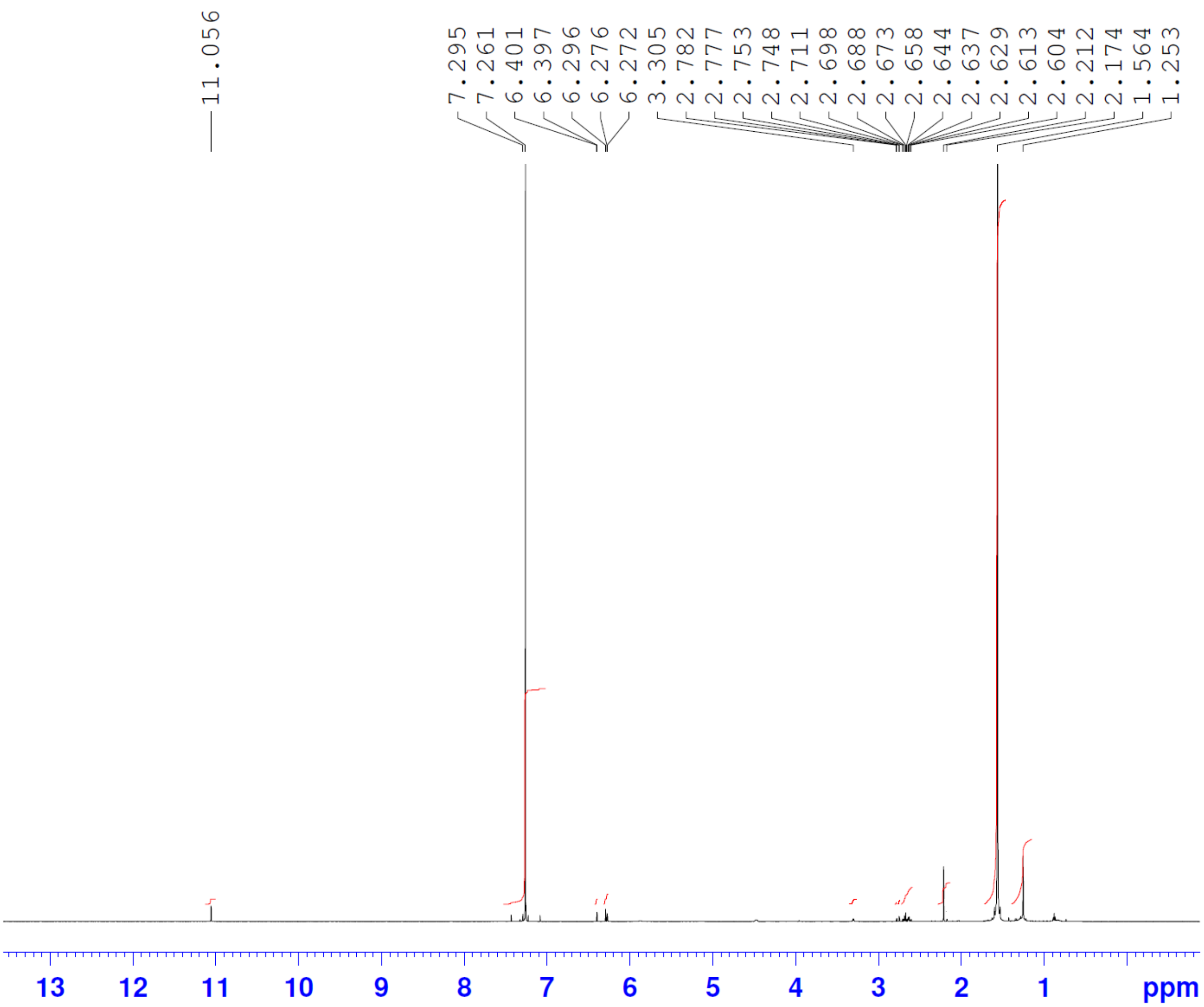

Figure S16. 600 MHz ^{1}H NMR spectrum of (+)-citreoisocoumarin (**3**) ($CDCl_3$) (Cont.).

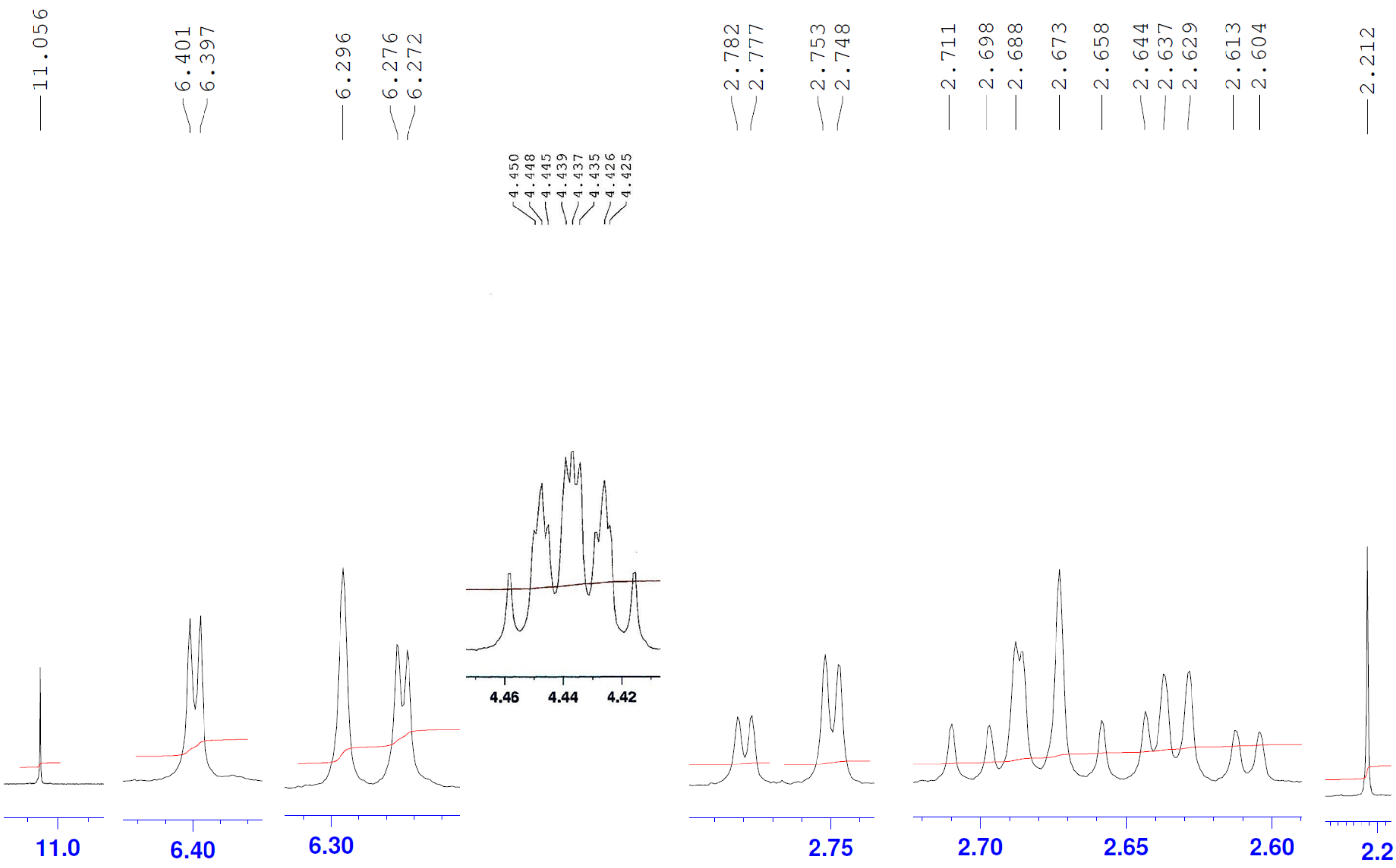

Figure S17. 150 MHz ^{13}C NMR spectrum of (+)-citreoisocoumarin (**3**) ($CDCl_3$).

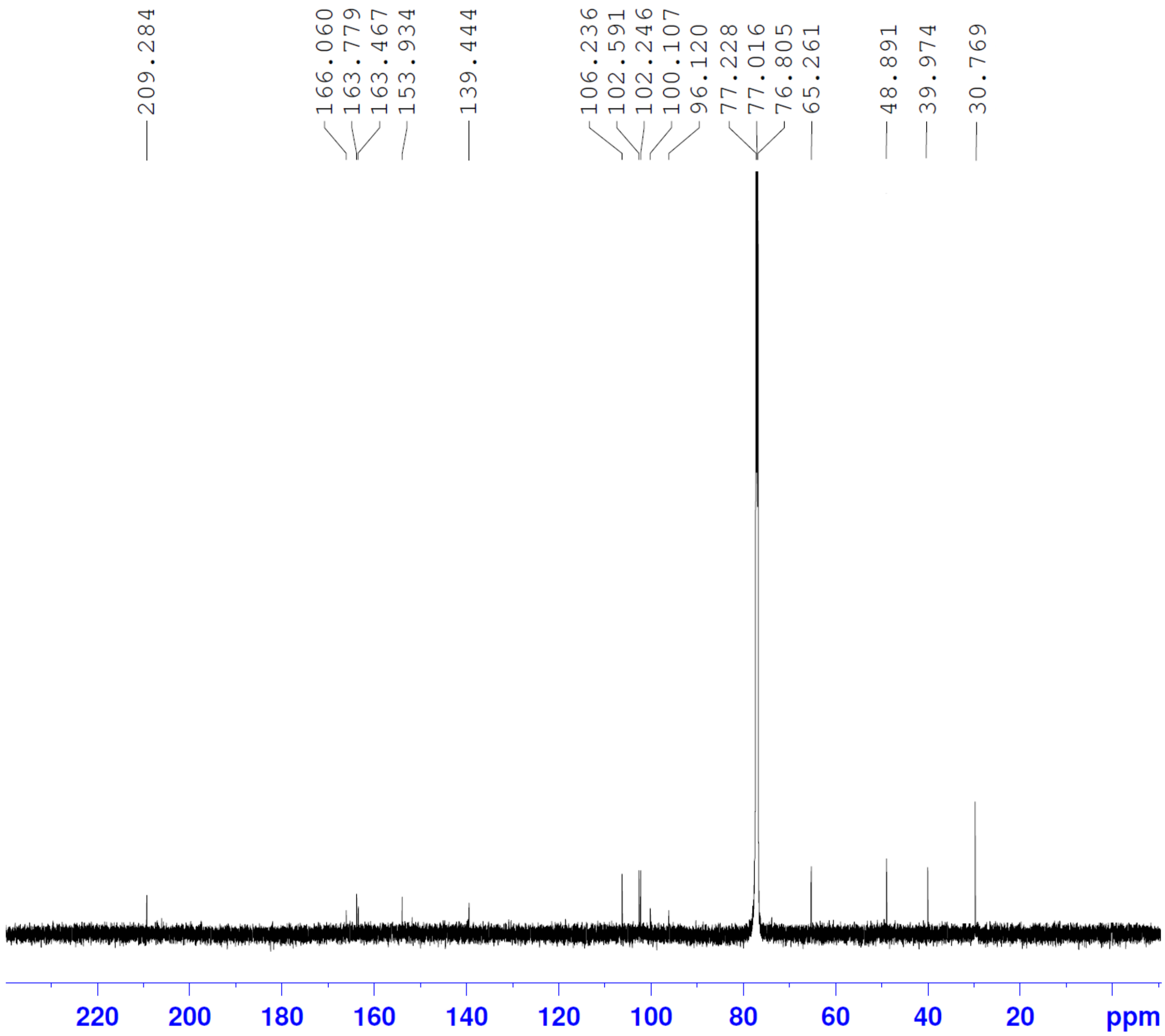

Figure S18. COSY spectrum of (+)-citreoisocoumarin (**3**) ($CDCl_3$).

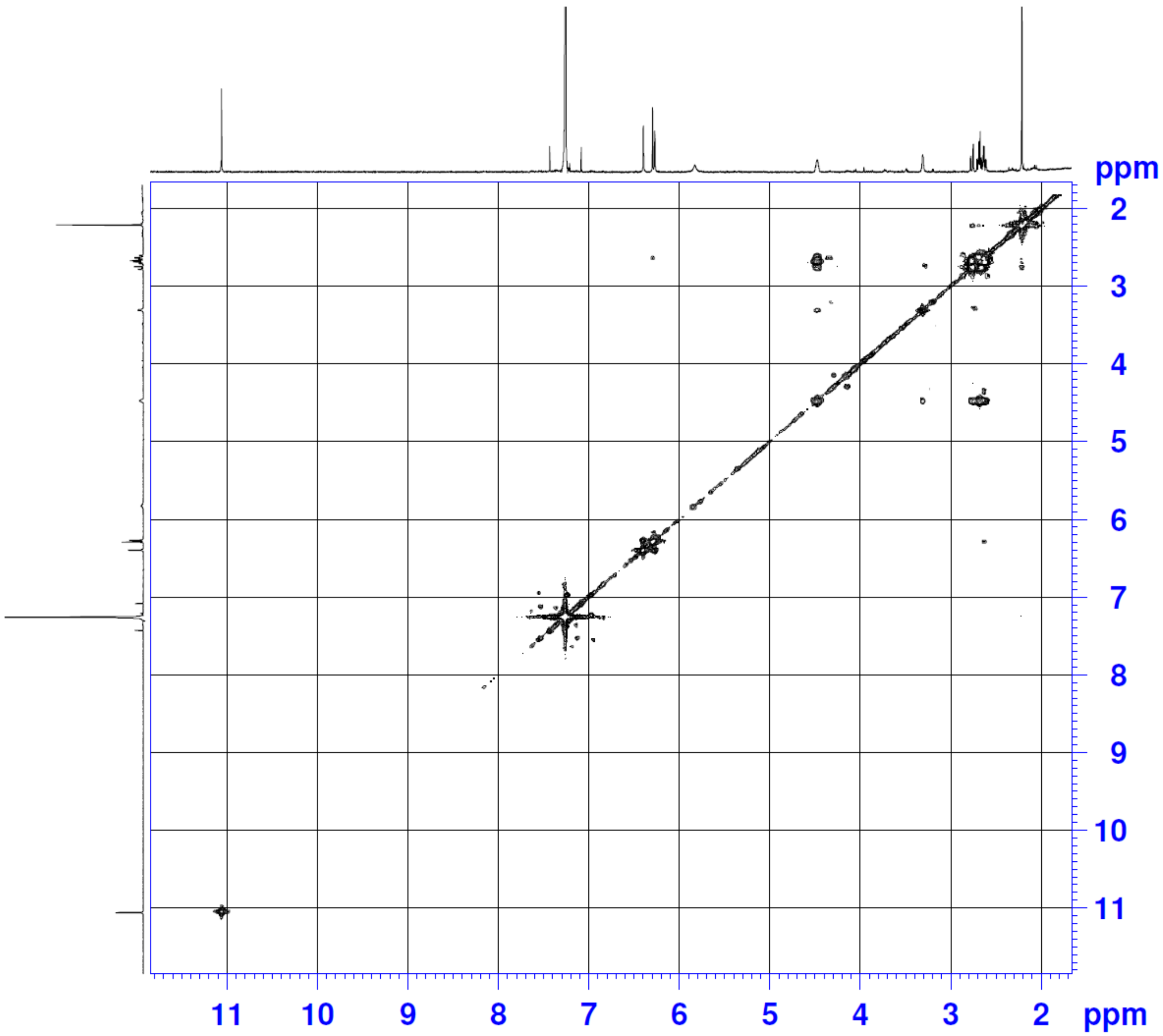

Figure S19. HSQC spectrum of (+)-citreoisocoumarin (**3**) ($CDCl_3$).

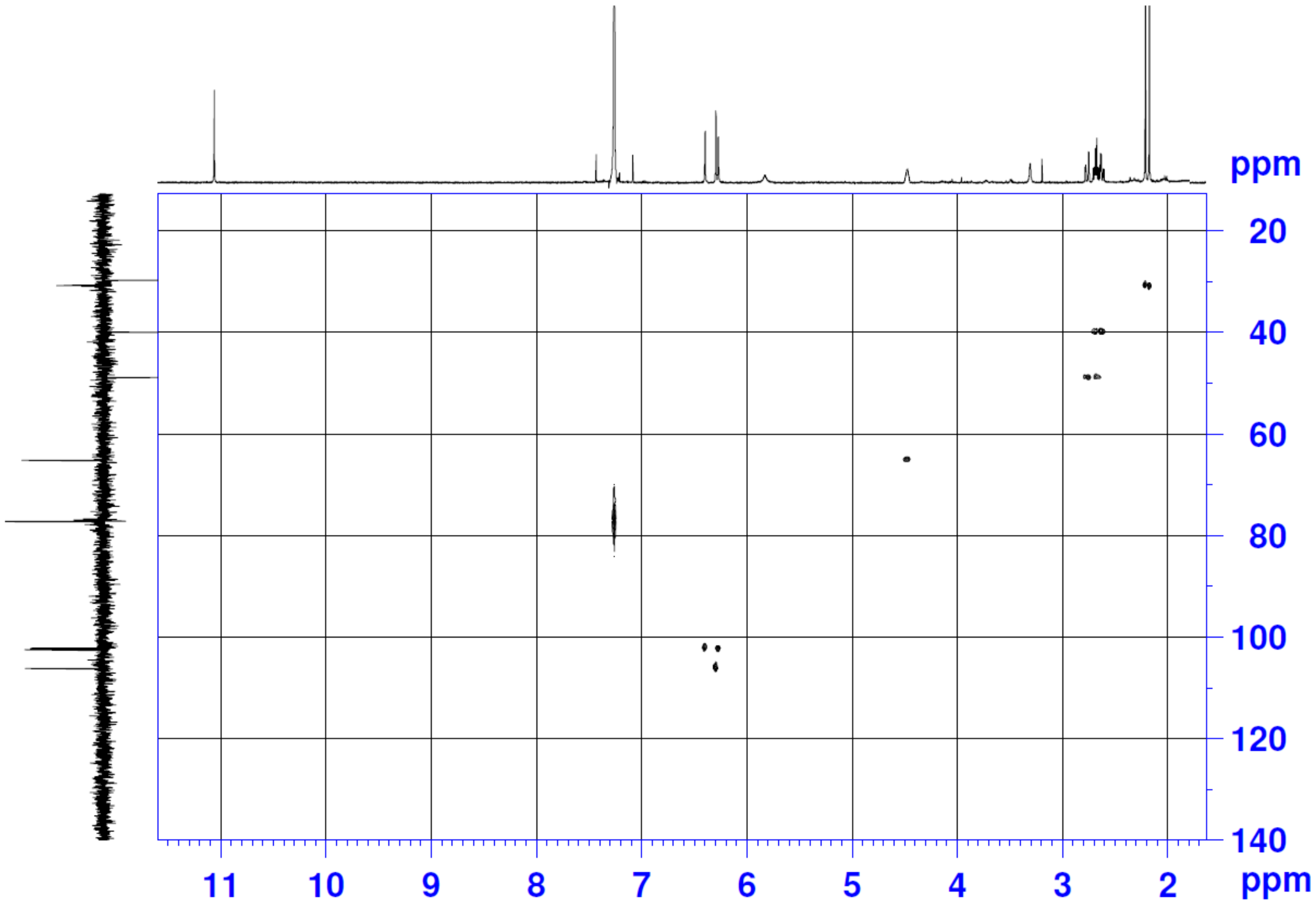

Figure S20. HMBC spectrum of (+)-citreoisocoumarin (**3**) ($CDCl_3$).

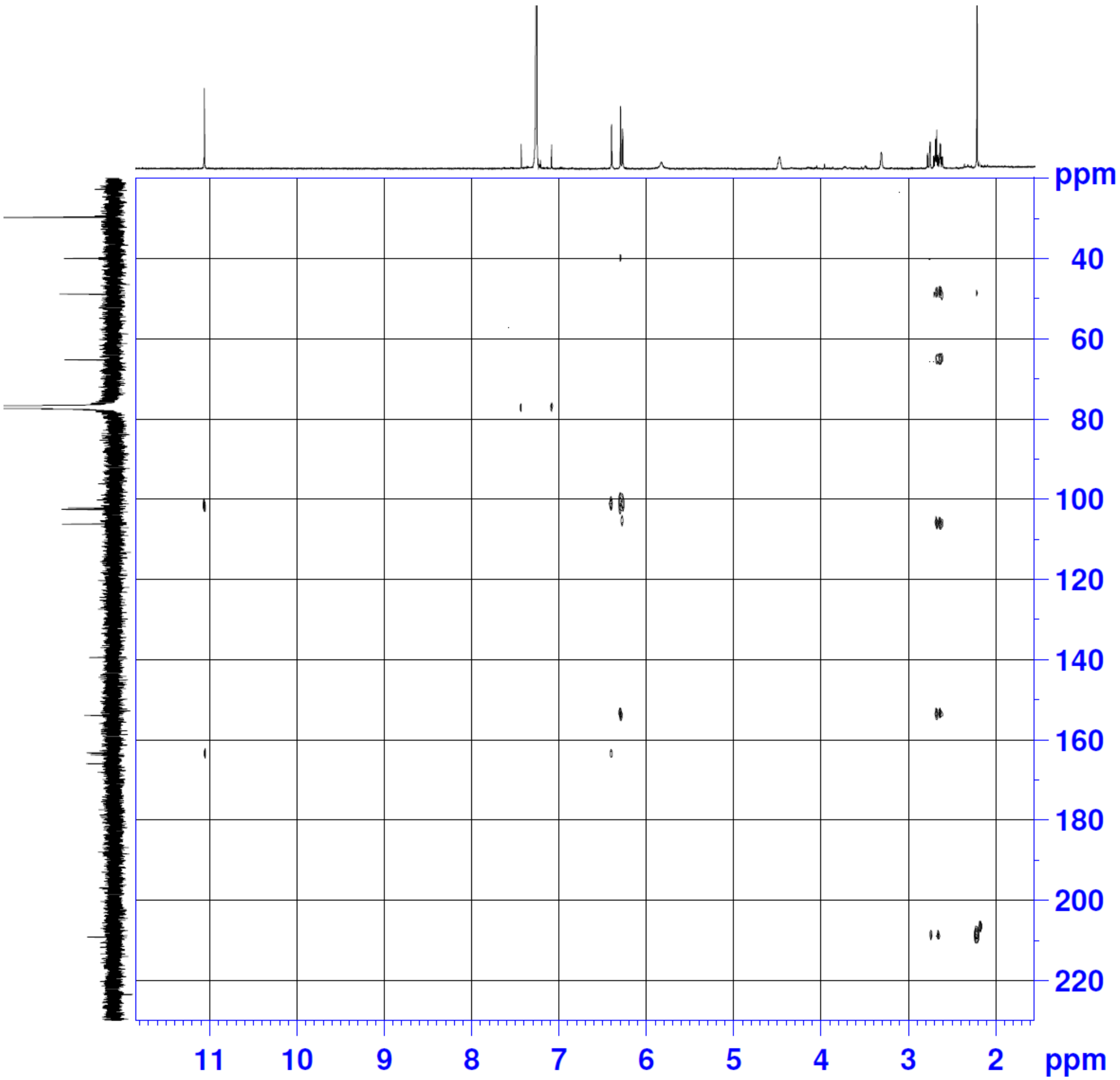

Figure 21. HRESIMS spectrum of (−)-6,8-di-*O*-methylcitreoisocoumarin (**4**).

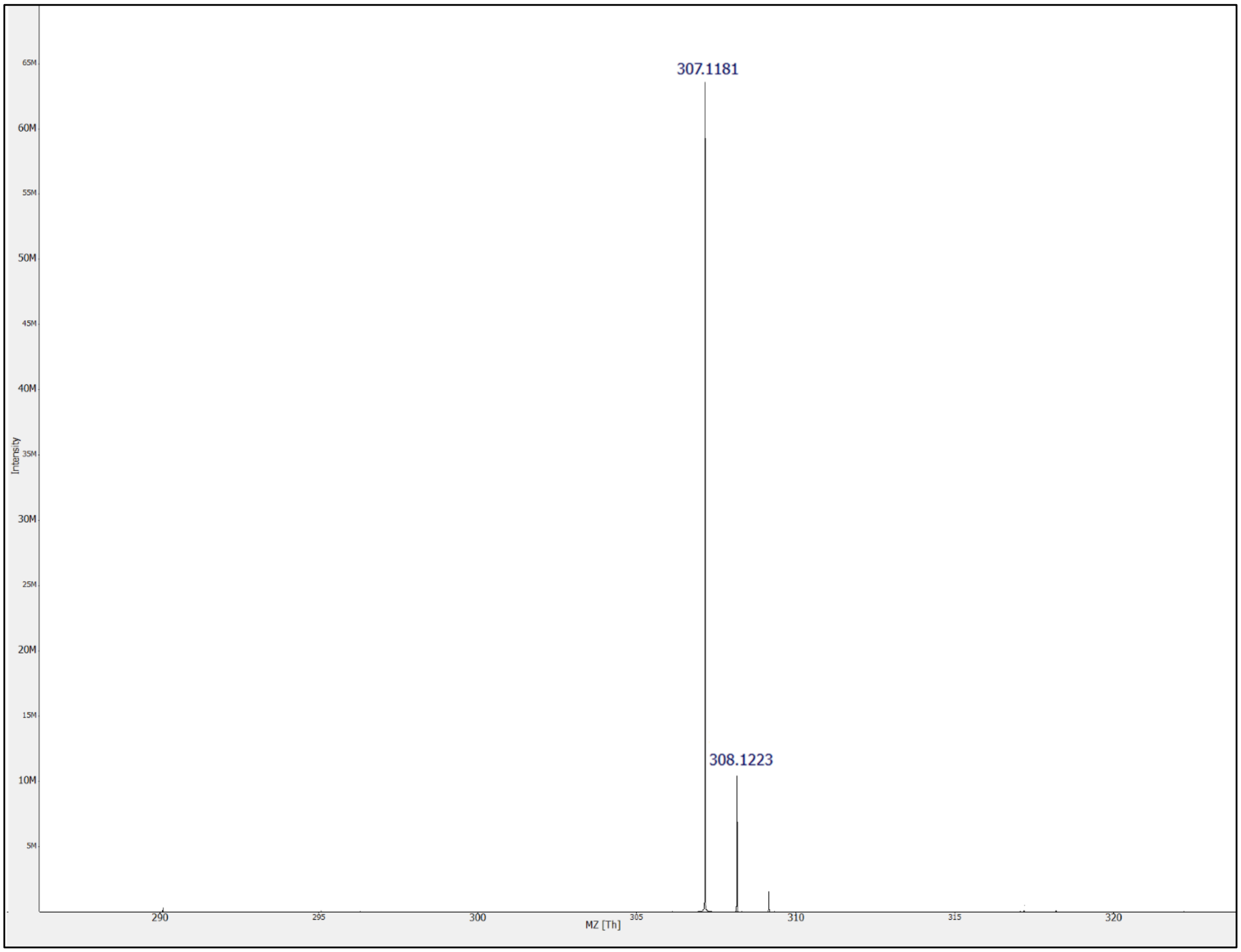

Figure S22. 600 MHz ¹H NMR spectrum of (−)-6,8-di-*O*-methylcitreoisocoumarin (**4**) ($CDCl_3$).

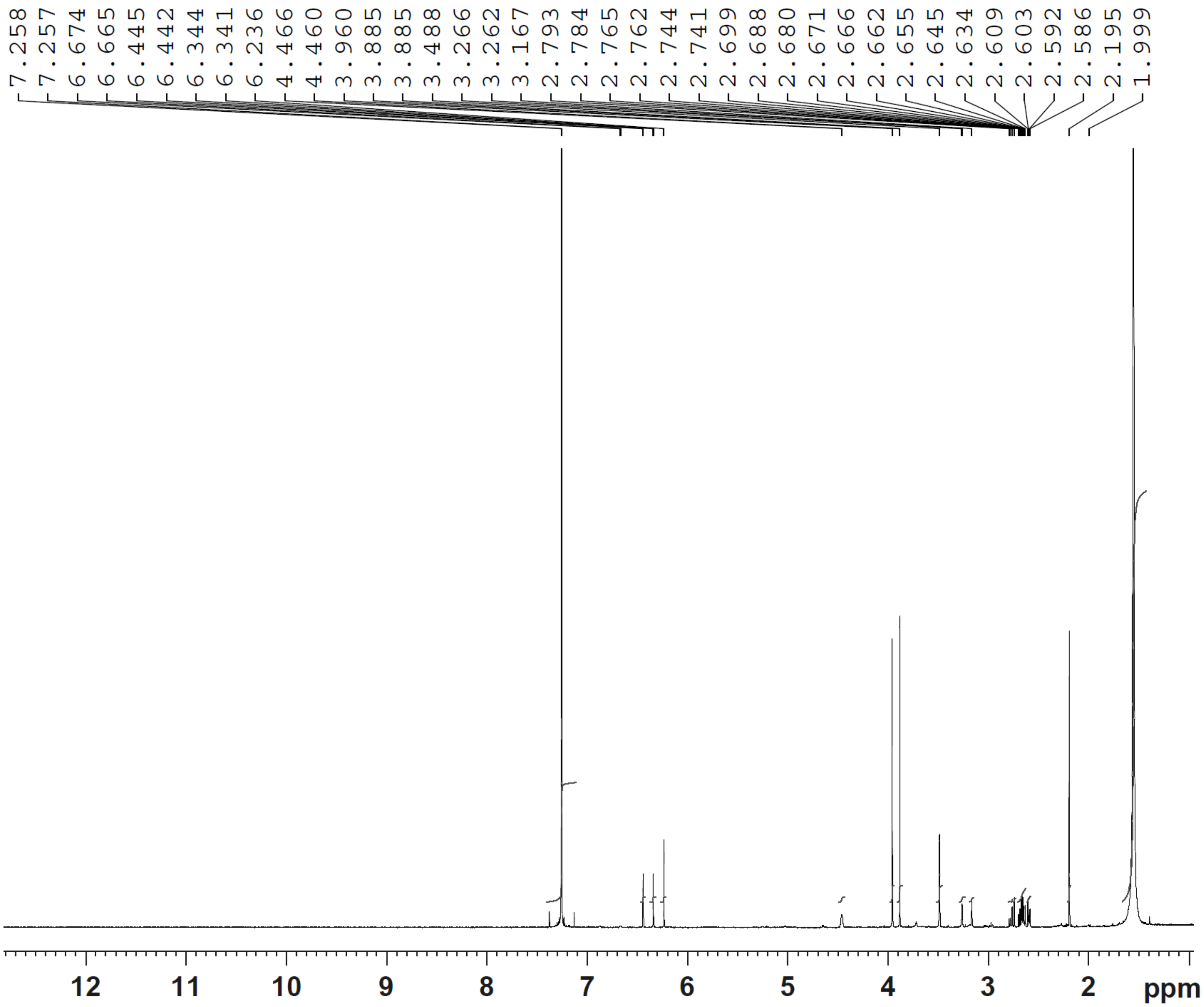

Figure S22. 600 MHz ^{1}H NMR spectrum of (−)-6,8-di-*O*-methylcitreoisocoumarin (**4**) ($CDCl_3$) (Cont.).

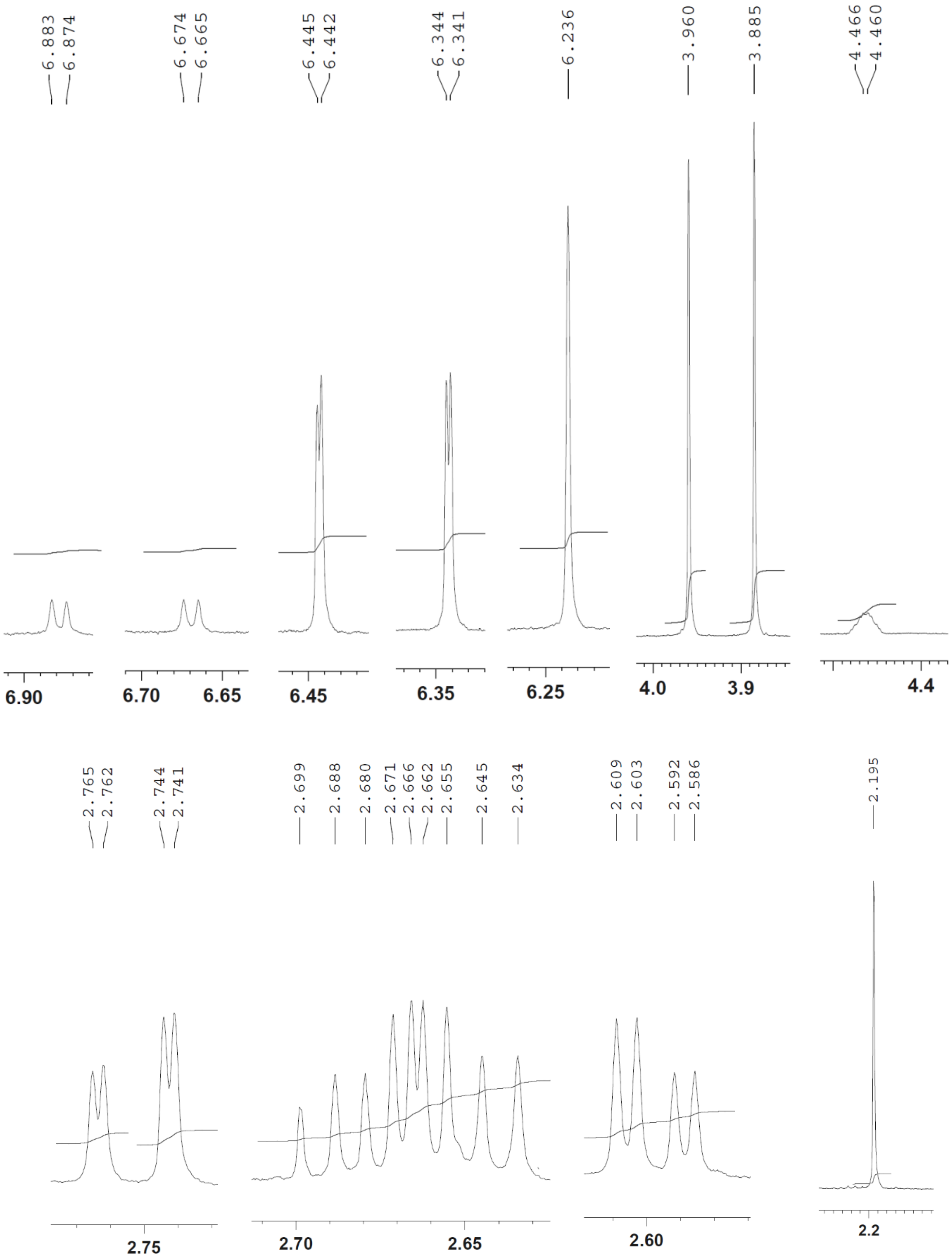

Figure S23. 150 MHz ^{13}C NMR spectrum of (−)-6,8-di-*O*-methylcitreoisocoumarin (**4**) ($CDCl_3$).

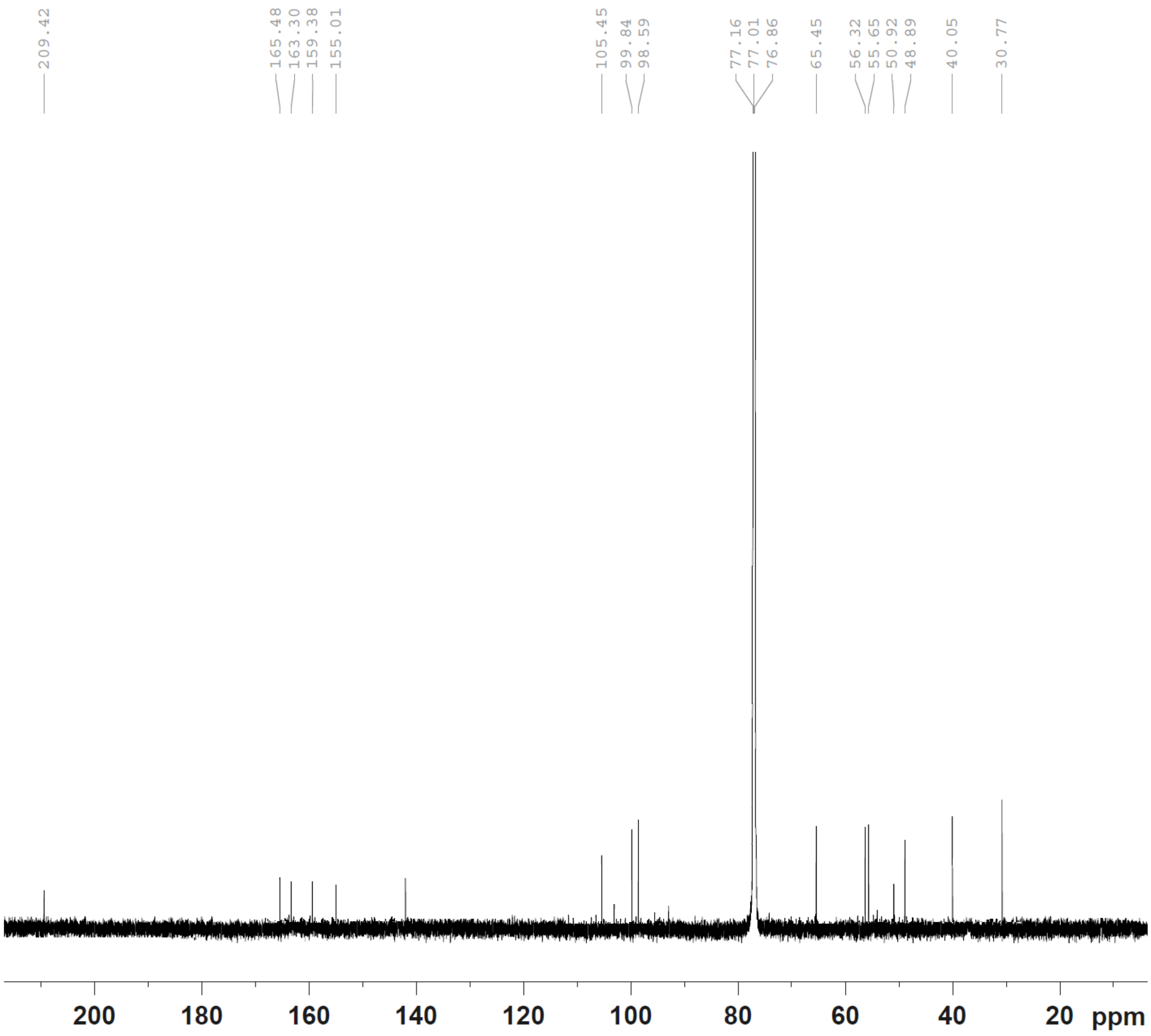

Figure S24. COSY spectrum of (−)-6,8-di-*O*-methylcitreoisocoumarin (**4**) ($CDCl_3$).

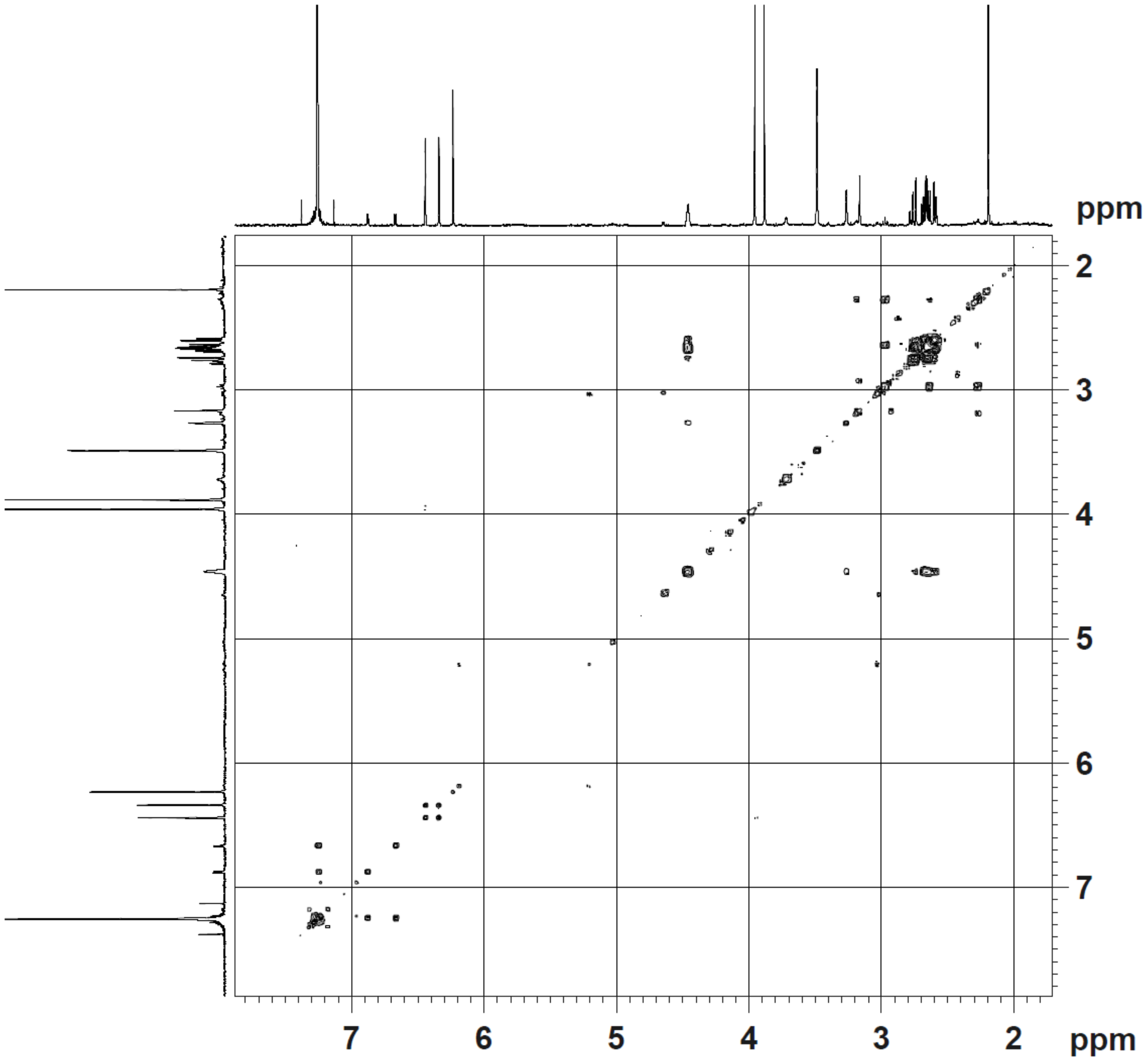

Figure S25. HSQC spectrum of (−)-6,8-di-*O*-methylcitreoisocoumarin (**4**) ($CDCl_3$).

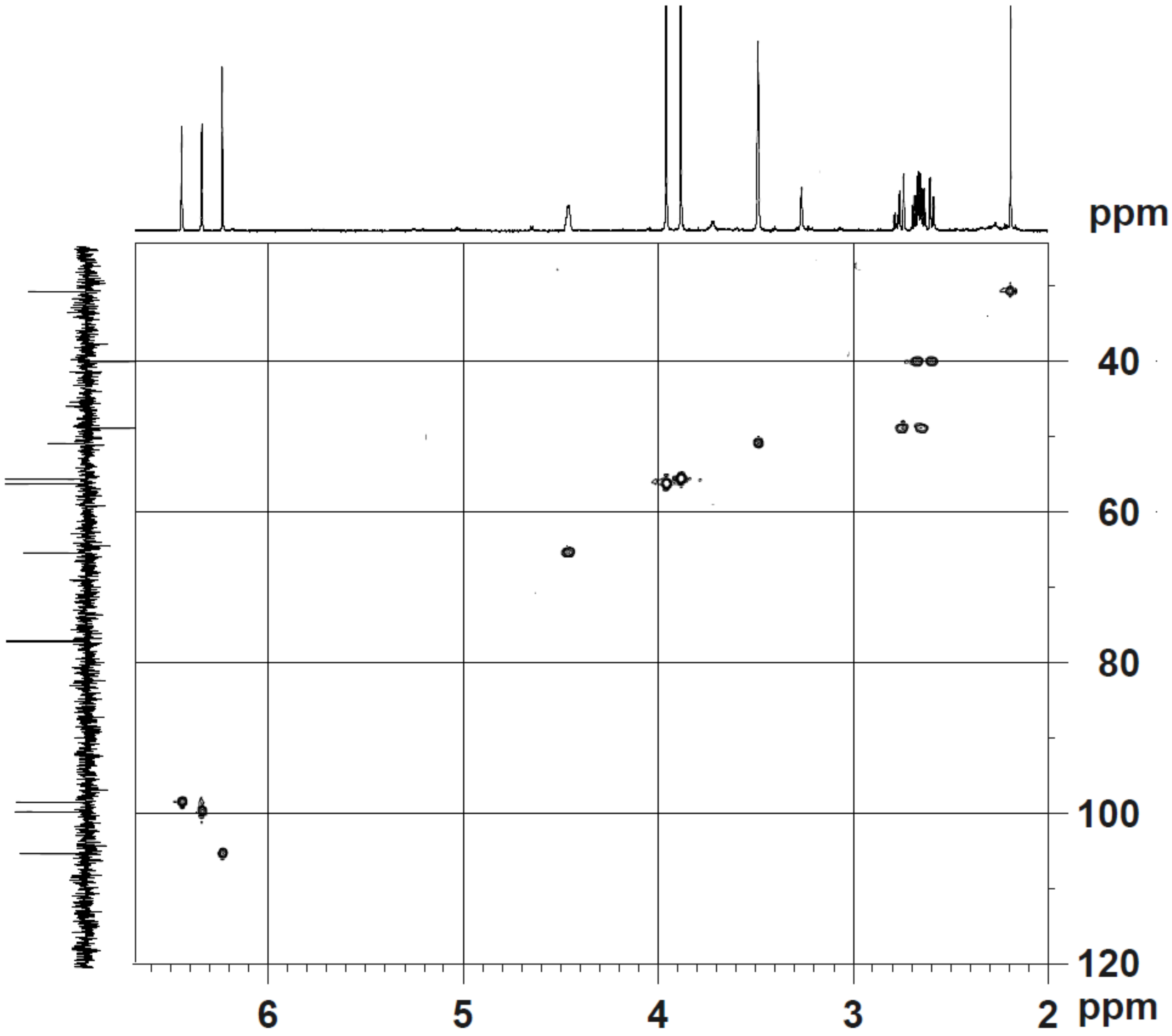

Figure S26. HMBC spectrum of (−)-6,8-di-*O*-methylcitreoisocoumarin (**4**) ($CDCl_3$).

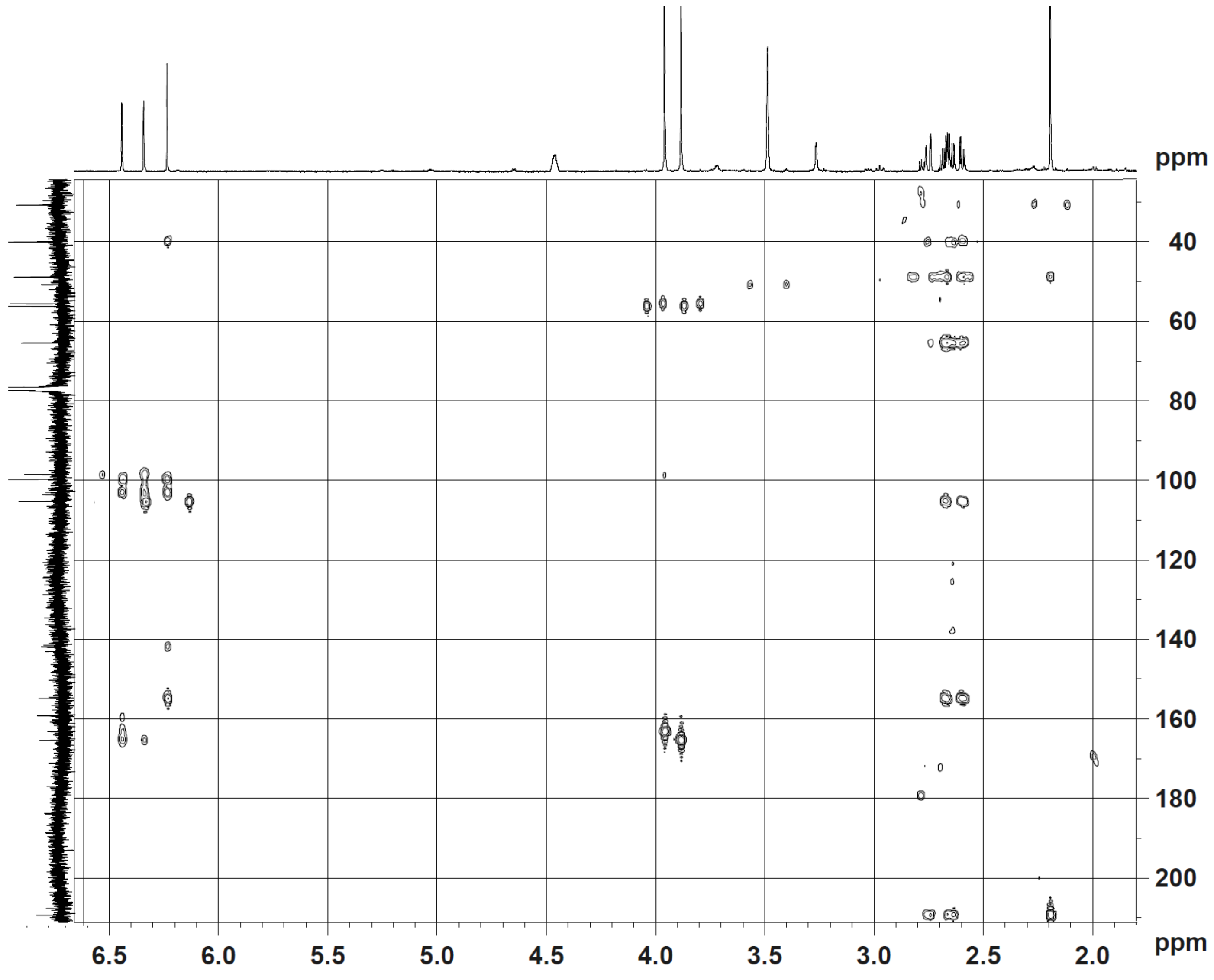

Figure 27. HPLC chromatograms of L-FDLA derivatized standard amino acid and the hydrolysates of compounds **1** and **2.**

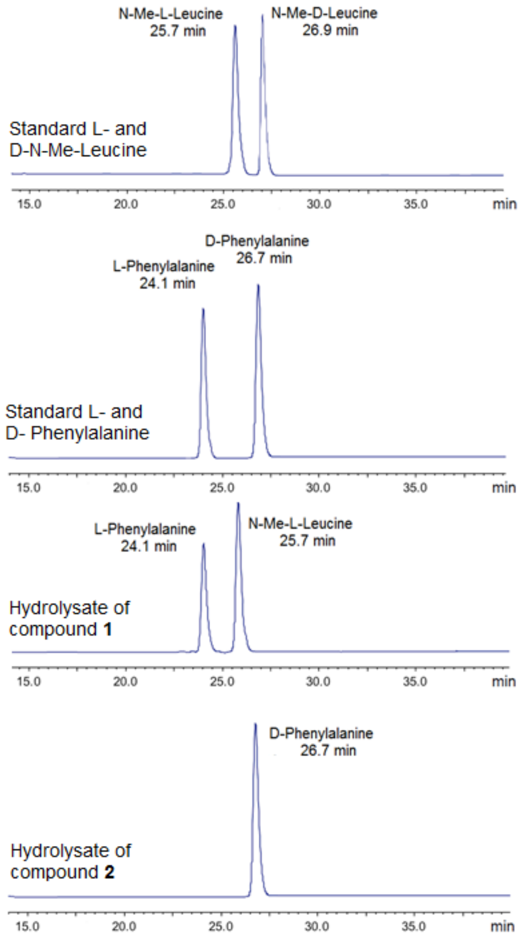

Supplement: Supplementary file 1 [file marinedrugs-20-00451-s001.zip › Asperopiperazines Aand B_Marine Drugs_2022_Supporting_R.pdf]
